# Integrative Studies of Human Cord Blood Derived Mononuclear Cells and Umbilical Cord Derived Mesenchyme Stem Cells in Ameliorating Bronchopulmonary Dysplasia

**DOI:** 10.3389/fcell.2021.679866

**Published:** 2021-11-09

**Authors:** Jia Chen, Yuhan Chen, Xue Du, Guojun Liu, Xiaowei Fei, Jian Ru Peng, Xing Zhang, Fengjun Xiao, Xue Wang, Xiao Yang, Zhichun Feng

**Affiliations:** ^1^The Second School of Clinical Medicine, Southern Medical University, Guangzhou, China; ^2^Department of Neonatology, Senior Department of Pediatrics, The Seventh Medical Center of PLA General Hospital, Beijing, China; ^3^National Engineering Laboratory for Birth Defects Prevention and Control of Key Technology, Beijing, China; ^4^Beijing Key Laboratory of Pediatric Organ Failure, Beijing, China; ^5^The First Affiliated Hospital of Dalian Medical University, Dalian, China; ^6^Shandong Qilu Stem Cell Engineering Co., Ltd., Jinan, China; ^7^Department of Neurosurgery, Xijing Hospital, Air Force Military Medical University, Xi’an, China; ^8^Department of Experimental Hematology and Biochemistry, Beijing Institute of Radiation Medicine, Beijing, China; ^9^Experimental Research Center, China Academy of Chinese Medical Sciences, Beijing, China

**Keywords:** bronchopulmonary dysplasia, cord blood mononuclear cell, umbilical cord mesenchymal stem cells, cytokines, whole transcriptome sequencing, noncoding RNA

## Abstract

Bronchopulmonary dysplasia (BPD) is a common pulmonary complication observed in preterm infants that is composed of multifactorial pathogenesis. Current strategies, albeit successful in moderately reducing morbidity and mortality of BPD, failed to draw overall satisfactory conclusion. Here, using a typical mouse model mimicking hallmarks of BPD, we revealed that both cord blood-derived mononuclear cells (CB-MNCs) and umbilical cord-derived mesenchymal stem cells (UC-MSCs) are efficient in alleviating BPD. Notably, infusion of CB-MNCs has more prominent effects in preventing alveolar simplification and pulmonary vessel loss, restoring pulmonary respiratory functions and balancing inflammatory responses. To further elucidate the underlying mechanisms within the divergent therapeutic effects of UC-MSC and CB-MNC, we systematically investigated the long noncoding RNA (lncRNA)–microRNA (miRNA)–messenger RNA (mRNA) and circular RNA (circRNA)–miRNA–mRNA networks by whole-transcriptome sequencing. Importantly, pathway analysis integrating Gene Ontology (GO)/Kyoto Encyclopedia of Genes and Genomes (KEGG)/gene set enrichment analysis (GSEA) method indicates that the competing endogenous RNA (ceRNA) network is mainly related to the regulation of GTPase activity (GO: 0043087), extracellular signal-regulated kinase 1 (ERK1) and ERK2 signal cascade (GO: 0070371), chromosome regulation (GO: 0007059), and cell cycle control (GO: 0044770). Through rigorous selection of the lncRNA/circRNA-based ceRNA network, we demonstrated that the hub genes reside in UC-MSC- and CB-MNC-infused networks directed to the function of cell adhesion, motor transportation (Cdk13, Lrrn2), immune homeostasis balance, and autophagy (Homer3, Prkcd) relatively. Our studies illustrate the first comprehensive mRNA–miRNA–lncRNA and mRNA–miRNA–circRNA networks in stem cell-infused BPD model, which will be valuable in identifying reliable biomarkers or therapeutic targets for BPD pathogenesis and shed new light in the priming and conditioning of UC-MSCs or CB-MNCs in the treatment of neonatal lung injury.

## Introduction

Bronchopulmonary dysplasia (BPD) is the most common complication associated with extremely preterm infants and is increasing in prevalence, most likely due to the increased survival of extremely low gestational age newborns (Thebaud et al., 2019). The pulmonary phenotypes of BPD are characterized by alveolar simplification, development retardation, impaired vascularization, progenitor cell reduction, as well as pulmonary function abnormality (Pasha et al., 2018). It was documented that 35% (18,000/50,000) of extremely preterm infants will develop BPD. Conventional therapies for BPD are symptom-targeted, whereas the mortality rate remains at high levels, with survivors displaying systematic adverse effects (Poets and Lorenz, 2018). Therefore, developing novel and efficient therapies to reduce overall morbidity and mortality in preterm infants with BPD is of great significance.

Stem cell-based therapies have been proven to alleviate various kinds of diseases, such as neurodegenerative diseases, heart malfunctions, as well as osteoporosis (Lou et al., 2021). Considering the self-renewal capacity, the multi-lineage differentiation, and site-directed mobilization, the broadly distributed mesenchymal stem cells (MSCs) have emerged as a key regulator in stem cell-based therapeutics for injuries (Thébaud, 2018). Recent data from allogeneic or autologous umbilical cord-derived MSC-based therapies have demonstrated promising results in studies based on animal models and early phase clinical studies of neonatal lung injury (Kang and Thébaud, 2018). Initially defined as adjuvant for human stem cell transplantation, mononuclear cells (MNCs) are another source of stem cell reservoir containing a high level of primitive multi-potent stem cells, progenitor cells, and regulatory T cells (Peters et al., 2015). MNCs consist of three categories, peripheral blood-derived mononuclear cells (PB-MNCs), bone marrow-derived mononuclear cells (BM-MNCs), and cord blood-derived mononuclear cells (CB-MNCs). Although a wide range of studies has revealed that MNCs with adult-appendage derivations are capable of presenting superior biological activity and regenerative efficacy in adult pulmonary conditions (Luan et al., 2012; Mills, 2017; Machado et al., 2018), conclusions on the effects of CB-MNCs toward treatment or pathogenesis of BPD remain vague. It is therefore worthwhile to determine the regulatory functions of human cord blood MNCs in an animal model. Furthermore, it is still hung in the balance whether a specific type of stem cell is the best candidate for a particular application in a certain disease model; comparison of the efficacies of UC-MSCs and CB-MNCs in preventing BPD is of great significance.

Noncoding RNAs (ncRNAs) are transcribed from more than 98% of human genome and regulate gene expression (Wright and Bruford, 2011), which can be subdivided into small noncoding RNAs [microRNAs (miRNAs)] and long noncoding RNAs (lncRNAs) as well as circular RNAs (circRNAs) by distinctive length and structure. Intriguingly, the reshaped expression profile of miRNA and lncRNA has been recently reported in the pathogenesis of a myriad of pathological and physiological conditions including BPD. For example, miR-206 (Duan et al., 2017) was boosted, whereas miRNA-489 (Olave et al., 2016) was shrunk in expression; the escalation of lncRNA–metastasis-associated lung adenocarcinoma transcript (MALAT)1 leads to the necrosis of type II alveolar epithelial cell (T2AEC), which ultimately induces lung injury (Cai et al., 2017). Strikingly, elevated levels of multiple miRNAs or lncRNAs have been found in preterm infants who later developed BPD (Syed et al., 2017; Chen J.H. et al., 2020), whereas most of the studies were conducted on cellular level or establishing a primitive correlation model in human specimens. Furthermore, circRNAs have attracted great research interest not only for its specific structure but also for its tissue- or developmental stage-specific expression features. Despite the accumulating knowledge obtained through studies from multiple human developmental diseases, reports about circRNA in the functional regulation of BPD are still poor.

Recently, the mechanisms of competing endogenous RNA (ceRNA) networks have been evidenced to explain a posttranscriptional layer of gene translation regulation. The classical ceRNA network is composed of various types of RNAs, such as lncRNAs, circRNAs, messenger RNAs (mRNAs), and pseudogenes. The potential regulatory lncRNA–miRNA–mRNA and circRNA–miRNA–mRNA pathways are therefore constructed on the basis of their shared bridge miRNAs imprinted with miRNA responsive elements (MREs). Although primitive studies have illustrated the putative circRNA-mediated ceRNA network in the pathogenesis of rat BPD model (Cheng et al., 2020), the comprehensive lncRNA–miRNA–mRNA and circRNA–miRNA–mRNA networks supported by physiological evidence are largely unknown. Moreover, priming and preconditioning of CB-MNCs or UC-MSCs with different cytokines or growth factors are of great importance in cell therapy-based treatment of BPD. Elucidation of the traceable biomarkers between stem cell implantation and BPD would suggest new strategies for combating this life-threatening disease.

In the current research, with the aim of assessing the efficiency of CB-MNCs and UC-MSCs in restoring lung function and balancing inflammatory responses in hyperoxia-induced BPD, we adopted an experimental mouse model to systematically evaluate the most appropriate cell infusion of indicated stem cells. We also attempted to elucidate the relevant inflammatory responsive mechanisms underlying stem cell-based therapies. Moreover, we adopted whole-transcriptome RNA sequencing (RNA-seq) to identify differentially expressed mRNAs (DEmRNAs), lncRNAs, circRNAs, and miRNAs. Kyoto Encyclopedia of Genes and Genomes (KEGG) and Gene Ontology (GO) pathway analyses were performed for differentially expressed RNAs (DE-RNAs) with significantly different expressions in BPD. Then, the ceRNA networks of mRNAs, lncRNAs, circRNAs, and miRNAs were constructed on the basis of evidence obtained from integrative miRNA target databases combined with the Pearson correlation analysis. Taken together, our findings may provide new evidence for the underlying mechanisms of ncRNAs and related ceRNA networks in stem cell-infused BPD and uncover novel targets for better utilizing stem cells in the treatment of BPD.

## Materials and Methods

### Study Approval and Ethics Statement

Animal procedures were reviewed and approved by the Animal Care and Ethics Committee of the Seventh Medical Center of PLA General Hospital (No. 2020-037). All animals were housed, cared for, and used in compliance with the guidelines regarding the humane use and care of laboratory animals for biomedical research published by the National Institutes of Health (No. 85-23, revised 1996).

### Mouse Model

Experimental male mice maintained on a C57BL6/J background were obtained from Beijing Vital River Laboratory Animal Technology and housed under pathogen-free conditions. Typically, a minimum of six mice were included in each treatment group, and all experiments were repeated at least three times.

For constructing the hyperoxia-based BPD model, newborn pups with both genders from different litters were pooled and then randomly distributed to exposure to room air (21% O_2_) and hyperoxia (85% O_2_) for 14 days (PN1–PN14) starting between 12 and 24 h after birth (recognized as PN1, postnatal day 1). Nursing dams were rotated between room air and hyperoxia every 24 h. Oxygen exposure occurred in an airtight plexiglass chamber equipped with an in-line oxygen analyzer and controller system (Jian-de Xin’anjiang Analysis Instrument Second Factory) in the same room as room air control animals. Oxygen concentrations were monitored continuously and maintained at 85% in the chamber during the experiment. Hyperoxia-exposed pups at PN7 were further randomly assigned to receive stem cell infusion. Briefly, CB-MNCs or UC-MSCs (3 × 10^6^ cells/kg, 0.03 ml) were delivered intravenously to pups through the great saphenous vein. Specifically, MSCs were transfected with green fluorescent protein (GFP)-tagged lentivirus to observe the distribution of cells. For blinding of the above experiments, mice were body-tagged with simple signs. The person who performed the experiments did not know the identity of the specific samples until data were collected and analyzed.

### Extraction and Characterization of Human Cord Blood-Derived Mononuclear Cells and Umbilical Cord-Derived Mesenchymal Stem Cells

Briefly, CB-MNCs and UC-MSCs were provided by Shandong Cord Blood Hematopoietic Stem Cell Bank. Human umbilical cord was sourced from uncomplicated full-terms, while cord blood was collected and cryopreserved from the punctured umbilical vein postpartum. All human-derived samples were collected following approval by the ethics review board of the Seventh Medical Center of Chinese PLA General Hospital.

For extraction of CB-MNCs, the cryopreserved cord blood units were thawed immediately and gently shook in 37°C water. Cord blood was collected into 50-ml centrifuge tube thrice volume of premixed suspension buffer within 5 min and stored at room temperature (RT). Mononuclear cells were isolated by centrifugation over a Ficoll-Hypaque density gradient at 700 rpm for 20 min at 4°C in premixed suspension buffer. Cells at the interface were collected by adding premixed suspension buffer followed by centrifugation at 500 rpm for 5 min at 4°C. The collected cells were washed thrice with phosphate buffered saline (PBS) and subsequently resuspended in serum-free Dulbecco’s modified Eagle’s medium (DMEM). The morphology of resuspended mononuclear cells was determined by Wright Giemsa staining.

Umbilical cord-derived mesenchymal stem cells extraction and purification were as described. Briefly, freshly collected UCs were washed with PBS three times and cut into segments. After removing the two arteries and one vein, the cord segments were cut into small pieces of approximately 1 mm^3^. The cord tissue blocks were cultivated in DMEM (Gibco, United States) supplemented with F12 and 10% fetal bovine serum (FBS; Gibco) in a humidified atmosphere at 37°C with 5% CO_2_ (Bu et al., 2017). Cells were subcultured once they attained 80% confluence. Medium was replaced every 2 days, and UC-MSCs at passage 5 were used for these experiments. Flow cytometry, Wright–Giemsa staining, alizarin red S staining, and oil red O staining were used to analyze the stem cell phenotype as revealed in Figure 2.

### Reagents and Antibodies

Antibodies were purchased from the following: primary antibodies subjected to immunohistochemistry including anti-vascular endothelial growth factor (VEGF)-α (Cat. #13034; 1:200), anti-matrix metalloproteinase (MMP)9 (Cat. #11132; 1:1,000), anti-transforming growth factor (TGF)-β (Cat. #11179; 1:1,000) were all purchased from Service Bio. Fluorescein isothiocyanate (FITC)-CD44 (Cat. #347943; 1:100), FITC-CD45 (Cat. #347643; 1:100), phycoerythrin (PE)-CD34 (Cat. #652802; 1:100), PE-CD73 (Cat. #550257; 1:100), PE-CD90 (Cat. #555896; 1:100), PE-CD105 (Cat. #580839; 1:100), HLADR (Cat. #555561; 1:100), PE mouse IgG1 (Cat. #349043; 1:100), and FITC mouse IgG1 (#349041; 1:100) for flow cytometry were purchased from BD Biosciences. For immunofluorescence, anti-human-CD44 (Cat. #5640; 1:1,600) were commercially bought from CST. The gradients of the premixed suspension buffer for CB-MNC extractions are commercially obtained as follows: PBS (Beijing Solarbio Science & Technology Co., Ltd.), 2% human albumin (Baxter International Inc.), and 10 U/ml heparin (Changzhou Qianhong Bio-Pharma Co., Ltd.).

### Immunofluorescence

Lung and brain tissue frozen sections were fixed in 4% paraformaldehyde 1 week post injection of indicated stem cells or sham controls. A standard immunofluorescence protocol was followed as previously described. Briefly, tissue was fixed in 1× zinc formaldehyde for 24 h at 4°C then rinsed with PBS. Tissue was dehydrated with a gradient of sucrose solution, cryo-sectioned, and rehydrated, followed by antigen retrieval. Blocking and staining were performed in 1% bovine serum albumin (BSA) in PBS supplemented with 0.3% Triton X-100. Sections were incubated in primary antibodies including mouse anti-CD44 (Cell Signaling no. 5640, used at 1:1,600) overnight at 4°C (Chen et al., 2014). The corresponding secondary antibodies were incubated with tissue for 1–2 h at RT. The nuclei were stained with 4′,6-diamidino-2-phenylindole (DAPI; Sigma), and images were captured and processed using identical settings in the Zeiss LSM 510 Meta inverted Confocal Microscope.

### Lung Morphology

Left-lobe lung sections (5 μm thick) were stained with H&E. For each morphometric analysis, 8–10 areas per slide were quantitated and averaged per slide. Images were acquired with a Nikon Eclipse TE300 inverted microscope, and quantification was performed using ImageJ. For radial alveolar count (RAC) measurement, the well-established method to quantify alveolarization (Hirsch et al., 2020), areas were randomly chosen and photographed at ×10 magnification. For each of six images, a perpendicular line was drawn from the center of a bronchial or bronchiolar airway to either the edge of the lung or the nearest connective tissue septum or airway. A minimum of 40 lines were drawn for each lung, and the number of septae intersected was counted for each line. Chord length (L_*m*_) of the airspace was estimated, as previously described (Cooney and Thurlbeck, 1982). Briefly, the images were superimposed on parallel on a grid with parallel lines spaced at 58-μm intervals, and the mean length of each chord, defined as the distance between two sequential intersections of the alveolar surface with the test line, was measured. For measuring the radical alveolar area, the Analyze Particles function of ImageJ was used in conjunction with a custom written macro for the measurement of the lung architecture and alveolar area (Nold et al., 2013). To prevent inadvertent observer bias, an investigator blinded to the assigned groups performed image acquisition and analyses. Values were pooled for each individual animal for statistical analysis.

### Transmission Electron Microscopy

Three slices of 2 mm × 2 mm × 2 mm were cut from three different segments of the left lung and fixed in 2.5% glutaraldehyde and phosphate buffer 0.1 M (pH = 7.4) for electron microscopy analysis. For each lung electron microscopy image (20/animal), the following alterations were analyzed as described previously (Buchacker et al., 2019) (a) alveolar-capillary membrane damage, (b) type II pneumocyte lesion, (c) type I pneumocyte infiltration, (d) elastic fiber breakdown, and (e) capillary and fibroblast deposition. Data were acquired using JEOL 1010 Transmission Electron Microscope, Tokyo, Japan.

### Quantitative Real-Time PCR

RNAs of lung tissue samples from indicated groups were reverse-transcribed into complementary DNAs (cDNAs) using the ReverTra Ace qPCR RT Kit (TOYOBO, OSAKA, Japan, FSQ-101), according to the manufacturer’s instructions. All primers used in the study are shown in Table 1. Real-time PCR was performed with THUNDERBIRD SYBR qPCR Mix (TOYOBO, OSAKA, Japan, QPS-201) on StepOnePlus^TM^ Real-Time PCR System (Roche Diagnostics, CA, United States, lightcycler 480). The PCR conditions were as follows: an initial denaturation step at 95°C for 5 min, followed by 40 cycles of 95°C for 10 s, and 60°C for 30 s. The results were analyzed using 2 ^–ΔΔΔ*CT*^.^ΔCT^ = _CT_ (target gene) − _CT_ (internal reference), ^ΔΔ^_CT_ = ^Δ^_CT_ (sample) − ^Δ^_CT_ (control). All experiments were performed in triplicate.

**TABLE 1:** Hematological Indicators.

Index	Control group	CB-MNCs group	*p*
WBC	4.80 ± 1.63	5.70 ± 1.99	0.3709
HGB	146.75 ± 8.88	130.88 ± 6.88	0.0022*
PLT	827.75 ± 137.77	741.38 ± 149.69	0.2802
PCT	0.47 ± 0.08	0.44 ± 0.08	0.3994
NEU#	0.22 ± 0.12	1.21 ± 0.95	0.0167*
LYM#	4.30 ± 1.48	3.30 ± 2.19	0.3358
MON#	0.25 ± 0.24	0.70 ± 0.64	0.1046
BAS#	0.01 ± 0.02	0.00 ± 0.00	0.2117
MON%	4.78 ± 5.10	13.53 ± 14.57	0.1558

*CB-MNC, cord blood-derived mononuclear cell; WBC, white blood cell (absolute numbers); HGB, hemoglobin; PLT, platelet (absolute numbers); PCT, Procalcitonin; NEU, neutrophils (absolute numbers); LYM, lymphocyte (absolute numbers); MON, monocyte (absolute numbers); MON%, percentage of monocyte; BAS, basophil (absolute numbers).*

*“#” means the absolute number of the index. Values are mean ±SD of a minimum of six animals in each group.*

*P < 0.05.

### Immunohistochemistry

Mice were anesthetized by intraperitoneal (i.p.) injection with 4% chloral hydrate (0.01 ml/g). Mouse lungs were collected after saline perfusion, and the tissue specimens were fixed overnight in 4% paraformaldehyde. After sequential steps of dehydration and embedding, 5-μm sections were rehydrated and stained with hematoxylin and eosin (Sigma-Aldrich) according to the manufacturer’s instructions. For immunohistochemistry, tissue sections were deparaffinized and incubated in citrate buffer at 95°C for 40 min for antigen retrieval and then incubated overnight at 4°C with the primary antibodies including anti-VEGFα (1:100), anti-MMP (1:100), and anti-TGF-β (1:100). After washing three times, tissue sections were incubated with biotinylated anti-rabbit IgG (1:200 dilution) for 1 h at RT after washing three times. Then, streptavidin–horseradish peroxidase conjugates were added, and the slides were incubated for 45 min. Here, 3,3′-diaminobenzidine (DAB) solution was added post PBS washing, and the slides were counterstained with hematoxylin. Negative controls were treated in the same way except without adding the primary antibodies. All staining was evaluated by a quantitative imaging method (Xie et al., 2014). Briefly, the percentage of immunostaining was calculated by immunohistochemistry (IHC) profiler plug-ins of the ImageJ software, and the staining intensity (negative, score = 0; weak, score = 1; medium, score = 2; very strong, score = 3) was recorded. An H-score was further calculated using the following formula: H-score = Σ (PI × I) = (percentage of cells of weak intensity × 1) + (percentage of cells of moderate intensity × 2) + (percentage of cells of strong intensity × 3). PI indicates the percentage of positive cells vs. all cells, and I represents the staining intensity.

### Pulmonary Function Assessment

For detection of respiratory motion function, mice were euthanized with 4% chloral hydrate (0.01 ml/g) i.p. followed by tracheostomy. Data were monitored and acquired by AcqKnowledge (Biopac Systems Inc., United States), a pulmonary maneuver system. During data collection, basic stable heart rate (HR) was recorded by ECG before tracheotomy operation. Mouse HR was maintained at proper level (<10% variation of basic HR) and breathing rate was maintained stable to ensure the reliability of the physiological data. Basic parameters included peak expiratory flow (PEF), peak inspiratory flow (PIF), breathing per minute (BPM), tidal volume (TV), and minute volume (MV).

For detection of pulmonary blood flow, all mice were subsequently transferred to evaluate pulmonary blood flow with laser Doppler flowmetry (LDF) using MoorFLPI (Moor Instruments, United Kingdom). Mice were ventilated (Alcott Biotech, China) with an average breathing frequency of 150 breaths/min, inspiratory/expiratory ratio 2.0, and tidal volume 1.0 ml/kg. The blood flow of bilateral lungs and heart were synchronizing measured after they were fully exposed. The pulmonary blood flow signal intensity was normalized to heart surface blood.

### Blood Sampling and Hematology Test

With the aim of evaluating the safety of CB-MNC infusion, hematological indicators were enrolled to present the overall physiological status. Briefly, a 1.5-ml ethylene diamine tetraacetic acid (EDTA)-coated eppendorf^®^ tube (EP tube) was used to collect approximately 200 μl of blood *via* the tail vein of the mice. The collected blood was placed at 4°C and transferred for analysis using BC-5180CRP (Mindray Instruments, Shenzhen, China).

### RNA Extraction and Library Preparation

Total RNA was isolated using TRIzol reagent (Invitrogen), according to the manufacturer’s instructions; RNA integrity was evaluated using the Agilent 2100 Bioanalyzer (Agilent Technologies, Santa Clara, CA, United States). The samples with RNA Integrity Number (RIN) R7 were subjected to the subsequent analysis of high-throughput sequencing. For small RNA-seq, a total of 1 μg of total RNA per sample was used for the small RNA library. Sequencing libraries were generated using NEBNext^®^ Multiplex Small RNA Library Prep Set for IlluminaR (NEB, United States) following the manufacturer’s recommendations. For mRNA+lncRNA sequencing, mRNA libraries were constructed using NEBNext^®^ UltraTM II RNA Library Prep Kit for Illumina^®^ (NEB Cat# E7770L/E7775L) following the manufacturer’s instructions.

### RNA Sequencing Analysis

mRNA–lncRNA seq reads were preprocessed as described previously (Chen W. et al., 2020). Briefly, the 150-nt paired-end retained reads were mapped to the reference genome (mice NCBI 37 assemblies) using STAR (version 2.5.3a) with the default parameters. The uniquely mapped reads with less than 2% mismatch were passed to StringTie (version 1.3.3b) for transcript assembly, and the fragments per kilobase of transcript per million mapped reads (FPKM) value was also generated for each gene. For the small RNA-seq reads, the clean reads with high quality were then aligned to the same reference genome using Tophat2 (version 2.0.13) with default parameters after FastQC assessment. miRNA reads were normalized with transcripts per million (TPM). Finally, the DEGs were called with limma and DEseq2 packages in R software with the criterion of an adjusted *p*-value < 0.1 as well as log2FC > 1. Notably, the *p*-values were attained by the Wald test and adjusted by BH method.

### Gene Ontology and Kyoto Encyclopedia of Genes and Genomes Pathway Analysis

Gene ontology analysis functionally associates DEmRNAs with GO categories, which consist of biological process (BP), molecular function (MF), and cellular component (CC) networks that attribute the gene subsets to defined terms^1^. KEGG pathway analysis is optimal for analyzing DEmRNAs with defined signaling pathways based on the latest KEGG^2^ database.

### Gene Set Enrichment Analysis

The association between DEmRNAs and hallmark molecular signatures was analyzed using gene set enrichment analysis (GSEA v2.2) as previously described. We use default settings to calculate the enrichment score (ES), which estimates whether a certain term of gene set from the Molecular Signatures Database (MSigDB) (here refers to the term “hallmark”) is enriched among the ordered predefined differently expressed gene sets or not. False discovery rate (FDR) < 0.05 was considered statistically significant.

### Strategies in Identification of ceRNA Pairs

Based on the expression levels of mRNAs, lncRNAs, circRNAs, or miRNAs, Pearson’s correlation coefficient and *p*-value were calculated for miRNA–target (mRNA/lncRNA/circRNA) or mRNA–lncRNA/circRNA coexpression networks. For miRNA–target, combining with evidence from miRNA–target-analyzing tools miRanda and Targetscan, negatively correlated pairs with Pearson’s correlation coefficient value < −0.9 and *p*-value < 0.05 were subjected to further analysis. mRNA–lncRNA/circRNA coexpression pairs with Pearson’s correlation coefficient value > 0.8 and p-value < 0.05 were retained. Subsequently, shared pairs from the predicted miRNA–target pairs from binding sites and the predicted pairs from the coexpression network were synergistically used for building ceRNA network. Finally, a hypergeometric test was introduced to filter the mRNA–miRNA–lncRNA/circRNA network of significance as previously described (Zhang et al., 2018).

### Network Building

**FIGURE 1:**
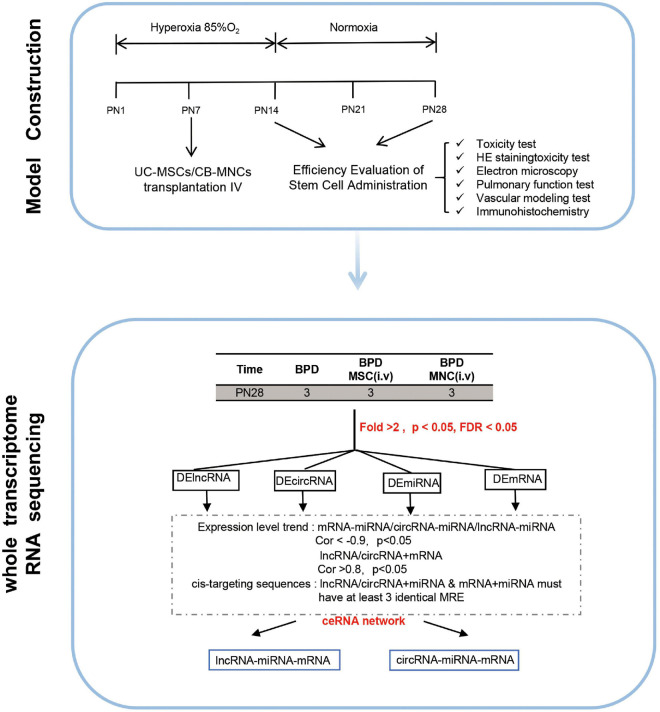
Graphic overview of the study design. The overall scheme of the current research is displayed. Newborn mice (C57 BL/6J) were exposed to hyperoxia (85% O_2_) for 14 days. Hyperoxia-exposed mice were compared with mice that remained in normoxic conditions (room air). Different indices and parameters evaluating the pulmonary–vascular functions were recorded and calculated. For better investigating the underlying mechanisms, whole-transcriptome sequencing was conducted in indicated groups. Details of the mRNA-seq, miRNA-seq, and circRNA-seq methods are described in the “Materials and Methods”.

Cytoscape software v.3.8.0 was utilized to construct and graph the corresponding networks (San Diego, CA, United States). In figures, distinct shapes of nodes define RNA types, and colors represent expression module. Patterns of edges define regulatory relationships. The size of the nodes represents the number of interactions (Figure 1).

### Statistical Analysis

Data were all mean ± SD. Comparisons between different groups were performed by one-way ANOVA followed by Bonferroni’s multiple comparison test or unpaired Student’s *t*-test (GraphPad v7.03; GraphPad Software Inc.). Statistical significance was defined as a two-sided *p*-value less than 0.05. All statistical analyses were graphed by Prism software program (version 7.03; GraphPad Software, San Diego, CA, United States). Data are representative of three independent biological replicates.

### Data Availability

Bioinformatics pipelines and scripts used for our analysis are available at https://github.com/mauve612/BPD-stem-cell-. All the datasets of RNA-seq included in this study have been uploaded to the Genome Sequence Archive at the National Genomics Data Center, Beijing Institute of Genomics (BIG), Chinese Academy of Sciences/China National Center for Bioinformation (GSA: CRA004720 with BioProject ID: PRJCA004041), and are publicly accessible at https://bigd.big.ac.cn/gsa/ after the release date of December 14, 2022.

## Results

### Characterization and Distribution of Umbilical Cord-Derived Mesenchymal Stem Cells (UC-MSCs) and Cord Blood-Derived Mononuclear Cells

With the aim of testing our hypotheses that the infusion of fetus-derived stem cell can alleviate the phenotype of BPD, we set out to establish a BPD model that can phenocopy the features of severe BPD as previously discussed (Nardiello et al., 2017). In this model, newborn mice were exposed to 85% O_2_ (hyperoxia) from postnatal day 1 (PN1) to PN14 and returned to room air at PN14 thereafter. Age-matched control litters were housed under standard room air conditions (normoxia) (Figure 1).

**FIGURE 2:**
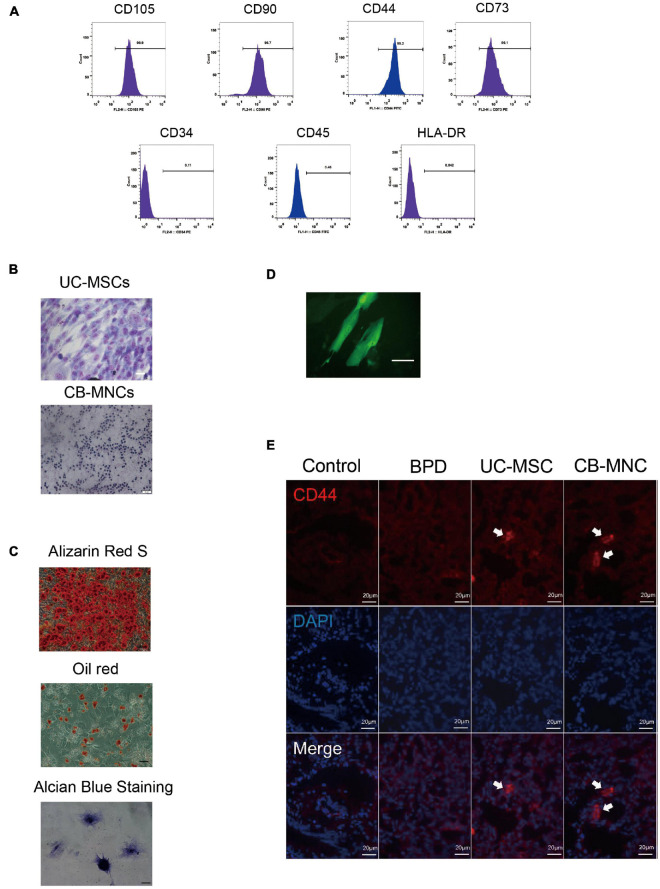
Intravenous infusion of cord blood derived-mononuclear cell (CB-MNC) and umbilical cord derive-messenchymal stem cell (UC-MSC) through great saphenous vein injection. **(A)** Surface marker expression of umbilical cord-derived mesenchymal stem cells (UC-MSCs). Flow cytometry analysis indicated that UC-MSCs were positive for CD105, CD90, CD44, and CD73 but negative for CD34, CD45, and HLA-DR. **(B)** Wright–Giemsa staining illustrating the purity of UC-MSCs (upper panel, scale bar: 50 μm) and cord blood-derived mononuclear cells (CB-MNCs) (lower panel, scale bar: 50 μm). **(C)** The lineage differentiation capacity of UC-MSCs was revealed by induction of distinct reagents. Alizarin red S for osteogenic capacity, oil red O for adipogenic capacity, and Alcian blue for chondrogenic differentiation, scale bar: 50 μm. **(D)** Fluorescent image indicating the successful infection of green fluorescent protein (GFP)-lentivirus in MSC, scale bar: 20 μm. **(E)** Immunofluorescence analysis of lung tissues infused with indicated cells. CD44 antibodies were used to detect human-derived CD44 (red), blue staining shows cell nuclei [4′,6-diamidino-2-phenylindole (DAPI)]. Images are representative of three independent experiments, scale bar: 20 μm. Data are representative of three independent biological replicates.

Before assessing the efficiency and safety of indicated stem cell infusion, purity of UC-MSCs and CB-MNCs was firstly characterized. Flow cytometry analysis of cell surface antigens revealed that UC-MSCs stained positive for the MSC markers CD73, CD44, CD90, and CD105 but negative for the hematopoietic lineage marker CD34, CD45, and the human leukocyte antigen HLA-DR (Figure 2A). Next, we observed the purities of UC-MSCs and CB-MNCs by Wright–Giemsa staining, as shown in Figure 2B. Randomly selected views have shown that the relative quantities of purified UC-MSCs (Figure 2B, upper panel) and resuspended CB-MNCs (Figure 2B, lower panel) are higher than 90%. Meanwhile, as shown by alizarin red S, oil red O, and Alcian blue staining, under specific culture conditions, UC-MSCs could differentiate into osteocytes and adipocytes *in vitro* (Figure 2C).

We went on to study the distribution of indicated stem cells after infusion. Initially, UC-MSCs were stably infected with GFP fluorescent virus, and intensive fluorescent signals were observed (Figure 2D). One day after stem cell infusion, GFP-labeled MSCs were found in the lung (Figure 2D), indicating the successful circulation of UC-MSCs. To further verify the residence of UC-MSCs and CB-MNCs, lung and brain sections of mice harvested at PN42 were stained with CD44, a human stem cell marker. UC-MSC- and CB-MNC-infused mice demonstrated strong immunofluorescent density, while tissue sections in BPD group can hardly detect any fluorescent signals in lung (Figure 2F). These results suggested that UC-MSCs and CB-MNCs were most prevalently residents in lung at indicated times post injection.

### Intravenous Infusion of Cord Blood-Derived Mononuclear Cells and Umbilical Cord-Derived Mesenchymal Stem Cells Improves Hyperoxia-Induced Bronchopulmonary Dysplasia

**FIGURE 3:**
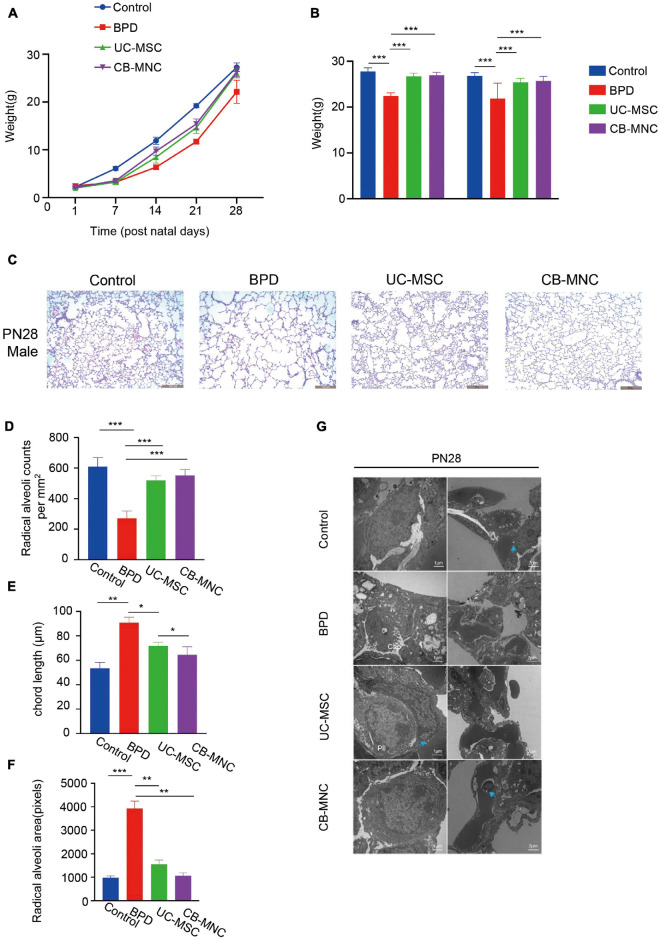
Intravenous infusion of cord blood-derived mononuclear cells (CB-MNCs) and umbilical cord-derived mesenchymal stem cells (UC-MSCs) improves hyperoxia-induced bronchopulmonary dysplasia (BPD). **(A)** Line graph illustrating the changes of weight in mice with different treatments at indicated time points. **(B)** Bar graph illustrating the change of weight in male and female mice with different treatments at postnatal day (PN)28. **(C)** Representative H&E staining images of lung histology in hyperoxia-exposed male mice with indicated treatments at PN28. Scale bar: 100 μm. **(D–F)** Sections of whole lungs were analyzed for the number of alveoli per square millimeter **(D)**, the chord length **(E)**, the size of the alveoli **(F)**. **(G)** Electron microscopy presenting the distal lung architecture of hyperoxia-exposed preterm mice subjected to different treatments at indicated time points. Pictures in left orientation indicated the type II pneumocytes, scale bar: 1 μm, whereas pictures in right orientation indicated the capillaries, scale bar: 2 μm. Values are mean ± SD of a minimum of six animals in each group, *p < 0.05, **p < 0.01, ***p < 0.001, by one-way ANOVA test. Data are representative of three independent biological replicates.

To explore the potential impacts on stem cell-implanted hyperoxia-induced BPD, the body weights of mice following stem cell infusion were again traced and recorded based on the time point of intense acute lung injury and active tissue remodeling. Strikingly, the growth rate of mice in UC-MSC and CB-MNC groups was significantly enhanced compared to that of the mice in BPD group, rendering the mouse weights of UC-MSC group and CB-MNC group comparable to that of control group at the endpoint of the observation (Figure 3A). Importantly, integrating sex and gender considerations into basic experimental biomedical research can largely improve the reproducibility and fidelity of the conclusion (Freeman et al., 2017), which have been adopted in previous animal studies regarding BPD model (Leary et al., 2019). No striking differences were observed between male and female mice in corresponding groups (Figure 3B). Mouse lung histology analyses were performed on mice of indicated groups at PN28 after injections. As indicated in Figure 3C, male animals subjected to infusion with CB-MNCs and UC-MSCs presented with dramatically improved alveolarization and almost completely restored lung architecture compared with those of the BPD group at both monitoring time points, and the conclusion was consistent in age-matched female animals (Supplementary Figure 1A), as reflected by an increase in radical alveolar counts (RACs), shrunk MIL (mean chord length, Lm), and the radical alveolar area (RAR) compared with normoxia-control mice at both genders (Figure 3D, Supplementary Figures 1B–D).

We also utilized electron microscopy to observe ultrastructure changes by hyperoxia exposure; gradual degeneration of the alveolar capillary membrane and damage to type II pneumocytes, higher septal barrier (see blue arrows, compare the annotated distances) and lower septal surface density, and reduced density of storage organelles were shown in BPD group compared to those of the controls (Figure 3G, upper two lanes). Significantly, both CB-MNC- and UC-MSC-infused group displayed ameliorated lung morphology. Interestingly, CB-MNC and UC-MSC infusion contributes to proliferation in type II pneumocytes lamellar bodies resembling those of control groups and repairs of alveolar capillary disorganizations (Figure 3G; lanes 3,4). Taken together, both CB-MNCs and UC-MSCs are capable of restoring lung morphology and improving pulmonary development of BPD mouse lung.

### Cord Blood-Derived Mononuclear Cells and Umbilical Cord-Derived Mesenchymal Stem Cells Attenuate Hyperoxia-Exposed Mouse Lung Inflammation Response

**FIGURE 4:**
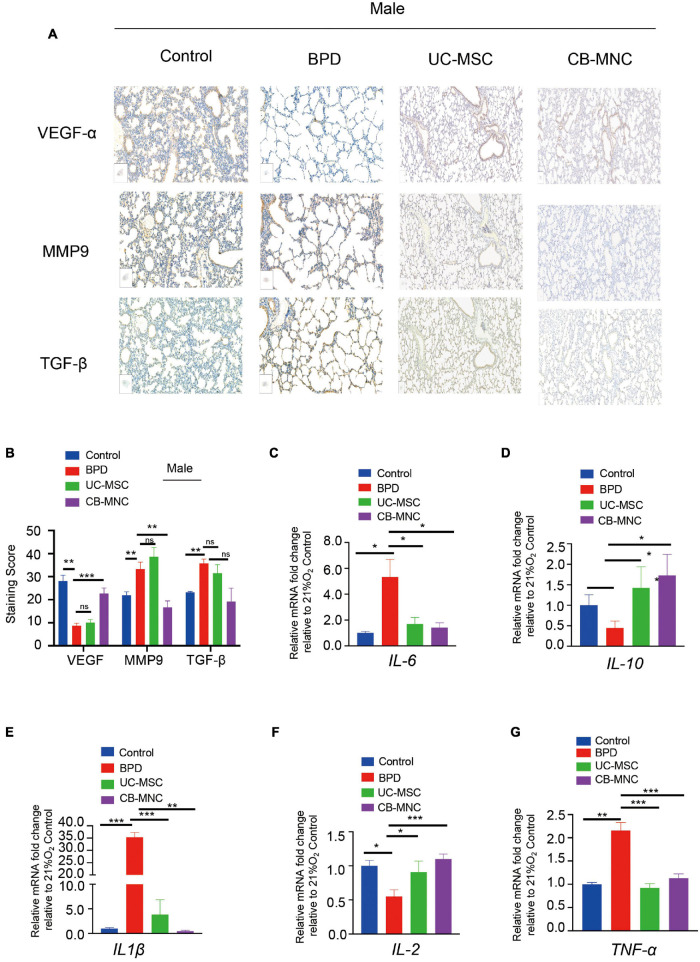
Expression profiles of inflammatory factors and in lung of UC-MSC- and CB-MNC-infused mice. **(A)** Immunohistochemical staining of vascular endothelial growth factor (VEGF), transforming growth factor-β1 (TGF-β1), and matrix metalloproteinase-9 (MMP-9) in lung tissue of male mice. Positive staining of cytoplasm is yellow-brown and that of nuclei is blue. Scale bar: 100 μm. **(B)** Summary of the quantification of the immunohistochemical staining of the indicated factors in **(A)**. **(C–G)** Lung tissue was collected from mice in each group at postnatal day (PN)28. Transcriptional levels of cytokines and growth factors were determined by quantitative reverse transcription polymerase chain reaction analysis. **(C)** Interleukin (IL)-6, **(D)** IL-10, **(E)** IL1-β, **(F)** IL-2, **(G)** tumor necrosis factor (TNF)-α. p-values are based on two-sided Mann–Whitney *U*-tests. *p < 0.05, **p < 0.01, ***p < 0.001. Data are representative of three independent biological replicates.

Given that inflammatory imbalance and abnormal growth are considered hallmarks of hyperoxia-induced BPD (Ryan et al., 2008), we examined the expression level of cytokines regarding vascular remodeling. As shown in Figure 4A, VEGF was significantly downregulated in BPD mice, which is consistent with the evidence that VEGF is decreased in infants dying with BPD and VEGF promotes lung angiogenesis and prevents alveolar damage in hyperoxia-exposed rats; injections of CB-MNCs restore their expression to the level comparable with the normoxia controls. TGF-β signaling plays a crucial role during lung development, and increased TGF-β levels negatively affect alveogenesis (Wu et al., 2020). Immunohistochemical staining analysis demonstrated that BPD mice had a marked increase in expression of the TGF-β and MMP-9 protein in the lung at 21 days of age compared to control group. Strikingly, there was significant abrogation in expression level of TGF-β1 and MMP-9 after CB-MNC infusion at both genders (Figures 4A,B and Supplementary Figures 2A,B). On the contrary, the expressions of TGF-β1 and MMP-9 were not significantly diminished post UC-MSC infusion compared to BPD mice among male mice. This might be due to a distinct mechanism of UC-MSCs and CB-MNCs that regulates the secretion of TGF-β1 and MMP-9 at different gender backgrounds of BPD. Subsequently, we adopted quantitative qPCR analysis to validate the expression levels of typical inflammatory factors and classical growth factors in lung tissue of hyperoxia-induced BPD mice, which were harvested 3 weeks post injection. We firstly examined the variations of the expression of indicated regulators in BPD model. Notably, there are folds of increase in expression of IL-6 and IL-1β concomitant with repression of IL-10, which is consistent with the long known evidence that elevated serum concentrations of pro-inflammatory cytokines IL-6 and IL-1β and declined anti-inflammatory IL-10 are markers of the pathogenesis of BPD in extremely low-birth weight infant (Yoon et al., 1997; Garingo et al., 2007). Infusion of UC-MSCs and CB-MNCs shrunk the expression of IL-6 and IL-1β, whereas it boosted the expression of IL-10 compared to BPD group (Figures 4C–E). In contrast to the sharp decline in BPD group, an increase of interleukin-2 (IL-2) was observed in the stem cell-infused groups (Figure 4F). IL-2 has been implied to play pivotal roles in T-cell activation and proliferation (Dhupkar and Gordon, 2017). We also observed that moderately decreased level of tumor necrosis factor-α (TNF-α) after UC-MSCs and CB-MNCs were infused (Figure 4G), which is supported by the evidence that preterm infants who went on to develop moderate or severe BPD showed significantly lower TNF-α levels at birth compared with no or mild BPD (Ehrhardt et al., 2016).

### Intravenous Infusion of Cord Blood-Derived Mononuclear Cells and Umbilical Cord-Derived Mesenchymal Stem Cells Improved Pulmonary Respiratory Motion

**FIGURE 5:**
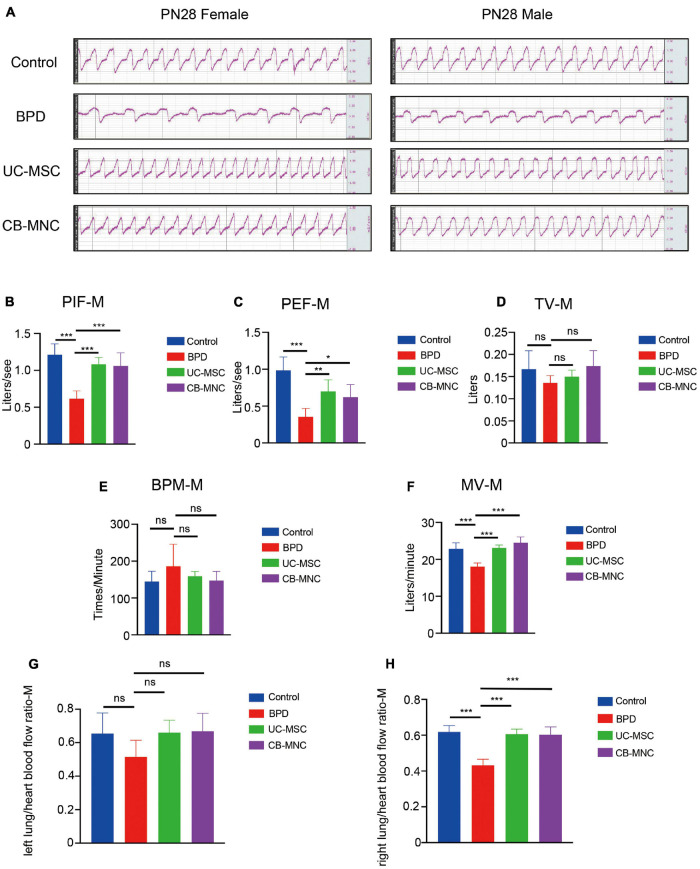
Intravenous infusion of CB-MNCs and UC-MSCs improved pulmonary respiratory function and pulmonary vessel circulation. **(A)** Representative respiratory waveforms of male and female mice at postnatal day (PN)28 after indicated treatments. **(B–F)** Pulmonary function testing among male mouse groups. The indicators include peak inspiratory flow (PIF) **(B)**, peak expiratory flow (PEF) **(C)**, tidal volume (TV) **(D)**, breathing per minute (BPM) **(E)**, and minute volume (MV) **(F)**. Values are mean ± SD of a minimum of six animals in each group,*p < 0.05, **p < 0.01, ***p < 0.001, by one-way ANOVA test). **(G,H)** Bar graph showing lung/heart blood flow ratio analysis of pulmonary vascular function in different groups at indicated time points. **(G)** Left side, **(H)** right side. Values are mean ± SD of a minimum of six animals in each group, *p < 0.05, **p < 0.01, ***p < 0.001, by one-way ANOVA test.

To elucidate the impact of CB-MNCs and UC-MSCs on improvement of respiratory motion, we conducted pulmonary function test using integrative pulmonary respiratory analyzing system. Parameters including peak inspiratory flow (PIF), peak expiratory flow (PEF), tidal volume (TV), breaths per minute (BPM), and minute volume (MV) were monitored and assessed in different groups of both genders at PN28. In accordance with the alterations in lung morphology, BPD mice displayed shortest breath and highest respiratory rate upon hyperoxia exposure, evidenced by the square respiratory waveform compared to those in controls (Figure 5A, lane 2). Interestingly, infusion of stem cells greatly reshaped the respiratory wave by shifting from square-like to sinusoidal (Figure 5A, upper two panels). Mice in stem cell-infused groups resulted in a significant escalation in multiple indices of respiratory waveform and decline in BPM and MV compared to those of BPD mice (Figures 5B–F, Supplementary Figures 4A–E). Strikingly, infusion of CB-MNCs exhibited even stronger capacity in restoring the respiratory motion function compared to those in UC-MSC-infused group.

### Intravenous Infusion of Cord Blood-Derived Mononuclear Cells and Umbilical Cord-Derived Mesenchymal Stem Cells Recovered Respiratory Blood Flow

With the aim of exploring the potential impact of UC-MSCs and CB-MNCs toward peripheral pulmonary vascular remodeling under hyperoxia exposure, lung perfusion with LDF was employed. Of note, left or right lung/heart blood flow ratio were recorded and calculated to analyze the pulmonary vascular resistance and pulmonary vascular areas. Notably, there was mild alteration of left lung/heart blood flow ratio after 3 weeks of infusion of UC-MSCs and CB-MNCs (Figure 5G, Supplementary Figure 4G), whereas the right lung/heart blood flow ratio were drastically escalated post UC-MSC and CB-MNC infusion (Figure 5H, Supplementary Figure 4H), compared to that of the BPD mice at both sex backgrounds. Collectively, these results suggested that stem cell injection, especially CB-MNC infusion, can improve the pulmonary vascular resistance and increased pulmonary vascular area upon hyperoxia stimulation.

### Toxicity Test

**FIGURE 6:**
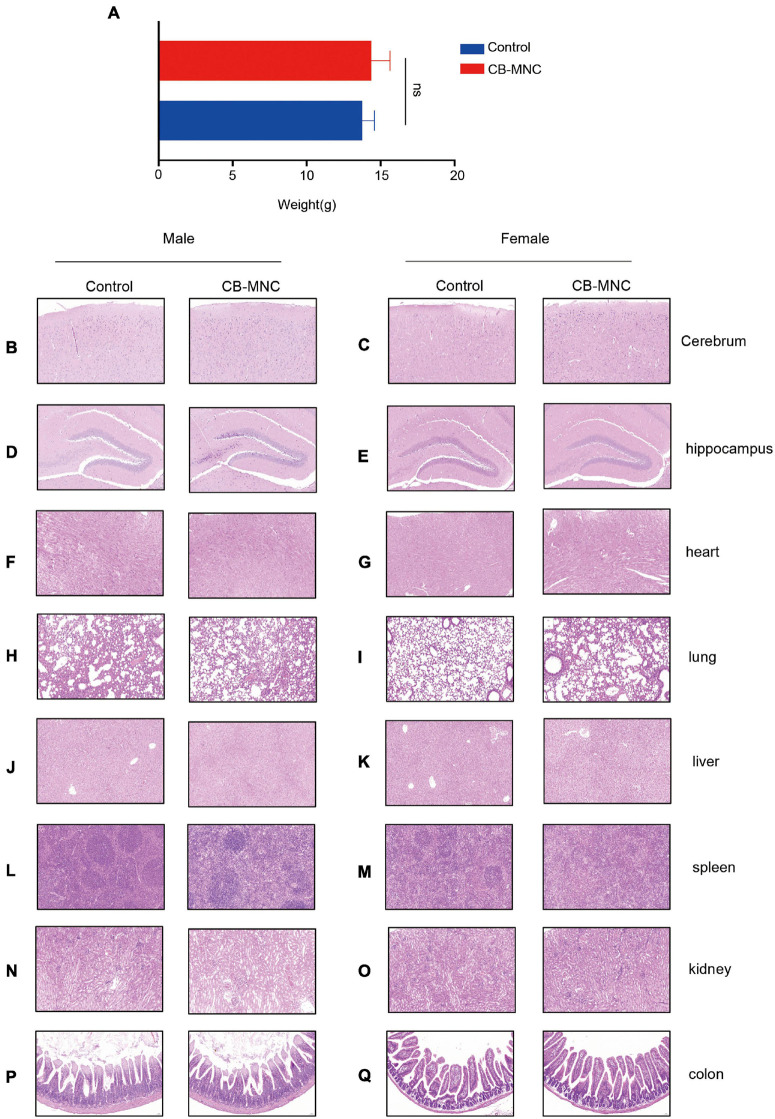
General report of pathology regarding toxicity test. Mice in control group and mononuclear cell (MNC) group [intravenous infusion 3 × 10^6^ cell/kg initiated at postnatal day (PN)7, twice in total] were sacrificed at PN28. Body weight was monitored **(A)**. At the end of the euthanasia infusion, neither cord blood-derived mononuclear cell (CB-MNC)-infused male nor female mice displayed significant abnormal changes in brain **(B–E)**, heart **(F,G)**, lung **(H,I)**, liver **(J,K)**, spleen **(L,M)**, kidney **(N,O)**, and colon tissues **(P,Q)**. Scale bar: 50 μm.

Current reports on safety application of CB-MNC of preclinical animal level are inadequate. We therefore assessed the toxic impact of CB-MNC infusion in C57 mice. During the experimental period, control group and BC-MNC group did not incur animal deaths. The body weight of animals in the CB-MNC group was not statistically different compared with that in the control group (Figure 6A). We also compared the hematological indicators in MNC-infused and control groups. Hematological indicators [hemoglobin (HGB) and lymphocytes (%)] were statistically significant (*p* < 0.05). Other hematological indicators white blood cells (WBC), platelet (PLT), neutrophils (NEUT) (×10^9^/L), lymphocytes (×10^9^/L), monocyte (MONO) (×10^9^/L), percentage of neutrophils [NEUT (%)], and percentage of monocyte [MONO (%)] were not statistically significant (*p* > 0.05) (Table 1). System autopsy at PN28 revealed no abnormal changes in the animals in indicated groups. There were no obvious pathological changes associated with the infusion of cells in the general and microscopic examinations, and representative histological images were shown in Figures 6B–Q. We observed unremarkable changes of corresponding parameters such as respiratory waveform, PIF, PEF, TV, BPM, and MV in CB-MNC group compared to those in control group at PN28 (Supplementary Figures 4A–E. The right or left lung/heart blood flow ratio of CB-MNC infusion was similar to those of the control group. Collectively, these results suggested that CB-MNC infusion will not influence the pulmonary vascular flow (Supplementary Figures 4F,G).

### Differentially Expressed Gene Analysis Between Bronchopulmonary Dysplasia and Stem Cell-Infused Groups

**FIGURE 7:**
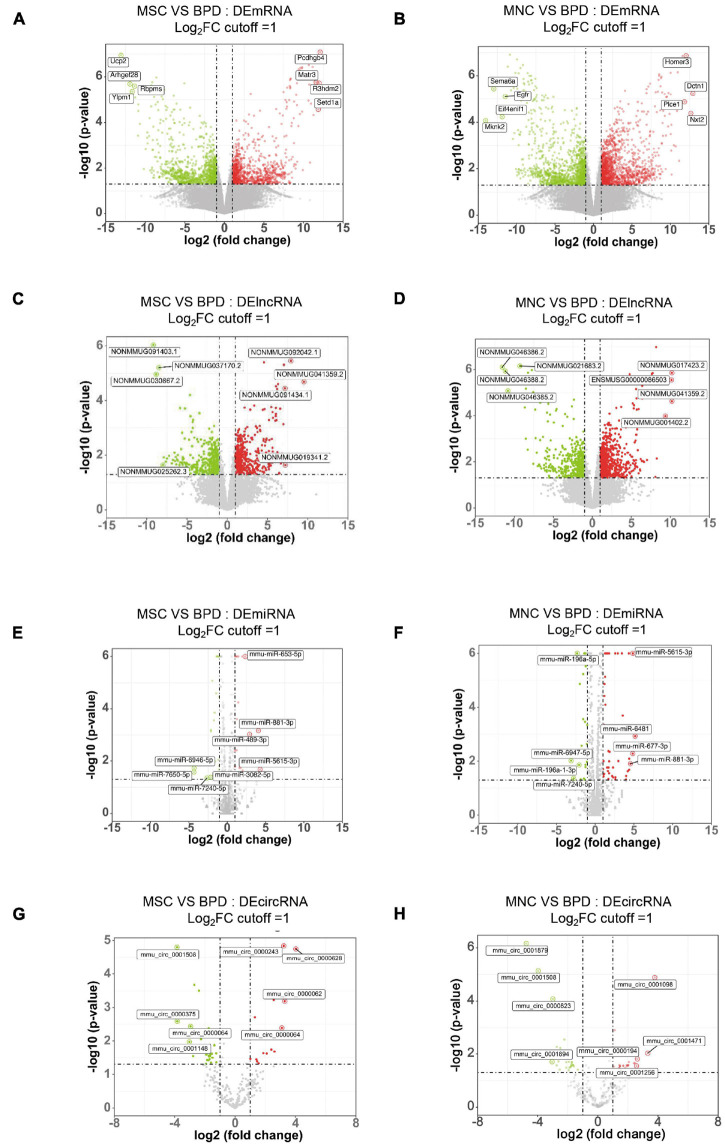
Expression profiles of distinct RNAs. **(A,B)** Expression profiles of mRNAs. In the volcano plots, red, green, and black points represent mRNAs that were downregulated, upregulated, and not significantly different in UC-MSC-infused mice **(A)** and CB-MNC-infused mice **(B)** relative to bronchopulmonary dysplasia (BPD) mice, respectively. x axis: log2 ratio of mRNA expression levels between stem cell-infused BPD mice and BPD mice. y axis: false discovery rate values (−log10 transformed) of mRNAs. **(C,D)** Expression profiles of lncRNAs. In the volcano plots, red, green, and black points represent circRNAs that were downregulated, upregulated, and not significantly different in umbilical cord-derived mesenchymal stem cells (UC-MSC)-infused mice **(E)** and cord blood-derived mononuclear cell (CB-MNC)-transplanted mice **(F)** relative to BPD mice. x axis: log2 ratio of lncRNA expression levels between stem cell-infused BPD mice and BPD mice. y axis: false discovery rate values (-log10 transformed) of circRNAs. **(E,F)** Expression profiles of circRNAs. In the volcano plots, red, green, and black points represent circRNAs that were downregulated, upregulated, and not significantly different in UB-MSC-infused mice **(E)** and CB-MNC-infused mice **(F)** relative to BPD mice. x axis: log2 ratio of circRNA expression levels between stem cell-infused BPD mice and BPD mice. y axis: false discovery rate values (−log10 transformed) of circRNAs. **(G,H)** Expression profiles of miRNAs. In the volcano plots, red, green, and black points represent miRNAs that were downregulated, upregulated, and not significantly different in UB-MSC-infused mice **(G)** and CB-MNC-infused mice **(H)** relative to BPD mice. x-axis: log2 ratio of miRNA expression levels between stem cell-infused BPD mice and BPD mice. y axis: false discovery rate values (-log10 transformed) of miRNAs.

As mentioned above, the therapeutic effects of CB-MNCs and UC-MSCs are divergent in trimming the imbalanced inflammatory network and restoring impaired structural remodeling. This phenomenon raised our attention to unravel the underlying molecular mechanisms with regard to the infusion of indicated stem cells. We therefore introduced whole-transcriptome sequencing, a highly popular and feasible approach, to analyze the differentially expressed (DE) ncRNAs (lncRNAs, circRNAs, miRNAs) and mRNAs in the compared groups. We adopted the value FPKMs to estimate the expression levels of mRNA transcripts. With cutoff of absolute Log2 fold changes ≥ 1 and adjusted p-value < 0.05, a total of 2,256 mRNA transcripts are significantly dysregulated, with 1,077 and 1,179 being, respectively, upregulated and downregulated in UC-MSC-transplanted mice relative to BPD mice (Figure 7A and Supplementary Table 1); 2,997 mRNAs were significantly dysregulated, with 1,595 and 1,402 being upregulated and downregulated in CB-MNC-infused mice relative to BPD mice (Figure 7B, Supplementary Table 1). After filtration of unannotated merged lncRNAs, we identified 1,065 significantly dysregulated lncRNA transcripts in UC-MSC-infused group relative to their levels in BPD mice group, within which 630 were upregulated and 435 downregulated (Figure 7C and Supplementary Table 2). As for CB-MNC-infused groups, 1,408 lncRNA transcripts were significantly dysregulated, 435 and 804 transcripts were upregulated and downregulated in CB-MNC-infused mice relative to BPD (Figure 7D and Supplementary Table 2). Next, based on TPM values, 42 miRNA transcripts were significantly dysregulated, with 21 and 21 being, respectively, upregulated and downregulated in UC-MSC-infused mice relative to BPD mice (Figure 7E and Supplementary Table 3); 69 miRNAs were significantly dysregulated, with 45 and 24 being, respectively, upregulated and downregulated in CB-MNC-transplanted mice relative to BPD mice (Figure 7F and Supplementary Table 3). Once again, differentially expressed circRNAs were revealed by spliced reads per billion mapping (SRPBM) value, and we mainly focus on the circbase-documented circRNAs for better validation of the functional markers. Here, 48 circRNA transcripts were significantly dysregulated, with 16 and 32 being, respectively, upregulated and downregulated, in UC-MSC-infused mice relative to BPD mice (Figure 7G and Supplementary Table 4); 58 circRNAs were significantly dysregulated, with 28 and 30 being, respectively, upregulated and downregulated in MNC-infused mice relative to BPD mice (Figure 7H and Supplementary Table 4). Detailed information on the number counts of the DEGs and the topmost DEGs was listed in Table 2; in UC-MSC-infused vs. BPD mice, the most upregulated mRNA was *Pcdhgb4* and the most downregulated mRNA was *Ucp2*. Besides, the most upregulated lncRNA, miRNA, and circRNA were NONMMUG041359.*2*, mmu-miR-5615-3p, and mmu_circ_0000628, respectively. The most downregulated lncRNA, miRNA, and circRNA were NONMMUG091403.1, mmu-miR-7650-5p, and mmu_circ_0000375, respectively. Whereas in groups between CB-MNC-infused and BPD mice, the most upregulated mRNA was *Nxt2* and the most downregulated mRNA was *Mknk2*. Besides, the most upregulated lncRNA, miRNA, and circRNA were NONMMUG017423.2, mmu-miR-6481, and mmu_circ_0001098, respectively. The most downregulated lncRNA, miRNA, and circRNA were NONMMUG046386.2, mmu-miR-6947-5p, and mmu_circ_0001879, respectively. We further analyzed the frequency distribution of the fold changes in different categories of DE-RNAs. As shown in Table 3, the 2–4-fold change (with Log_2_FC 1∼2) was most common in all kinds of RNA transcripts between UC-MSC and CB-MNC injection group, while the percentages of differentially expressed mRNAs, lncRNAs, and circRNAs among Log_2_FC ranging 2∼3 as well as 3∼4 were significantly higher in CB-MNC-infused group than that of the UC-MSC-infused group, which was in opposite trend with distribution of miRNA.

**TABLE 2:** Statistical analysis of all of the differently expressed ncRNAs and mRNAs.

DE-RNAs	Total No.		Up No.		Down No.		The most upregulated		The most downregulated	
							(log2 Fold change)		(log2 Fold change)	
	MSC BPD	MNC BPD	MSC BPD	MNC BPD	MSC BPD	MNC BPD	MSC/BPD	MNC/BPD	MSC/BPD	MNC/BPD
mRNA	2,256	2,997	1,077	1,595	1,179	1,407	*Pcdhgb4 (11.85)*	*Nxt2 (10.87)*	*ucp2* (−13.07)	*Mknk2* (−14.17)
lncRNA	1,065	1,408	630	804	435	604	NONMMUG0 41359.2 (10.02)	NONMMUG 017423.2 (10.20)	NONMMUG 091403.1 (−9.18)	NONMMUG 46386.2 (11.56)
miRNA	42	69	21	45	21	24	mmu-miR-5615-3p (4.28)	mmu-miR-6481 (3.44)	mmu-miR-7650-5p (−2.34)	mmu-miR-6947-5p (−4.32)
circRNA	48	58	16	28	32	30	mmu_circ_00 00628 (4.02)	mmu_circ_0 001098 (3.87)	mmu_circ_0 000375 (−3.85)	mmu9_circ_0 001879 (−4.76)

**TABLE 3:** Summary of distribution of indicated DE-RNAs.

Log2 Fold Change (statistics)	mRNA		lncRNA		miRNA		circRNA	
	MSC	MNC	MSC	MNC	MSC	MNC	MSC	MNC
>4	36.25	37.85	15.86	15.79	9.00	11.54	2.08	2.72
1∼2	43.71	37.60	62.54	59.56	71.42	75	54.17	49
2–3	12.1	15.76	12.6	14.64	19.04	9.62	29.17	31.38
3∼4	7.93	8.79	8.64	10.01	0.00	3.85	14.58	16.90

### Pathway Analysis in Differentially Expressed ncRNAs and mRNAs

**FIGURE 8:**
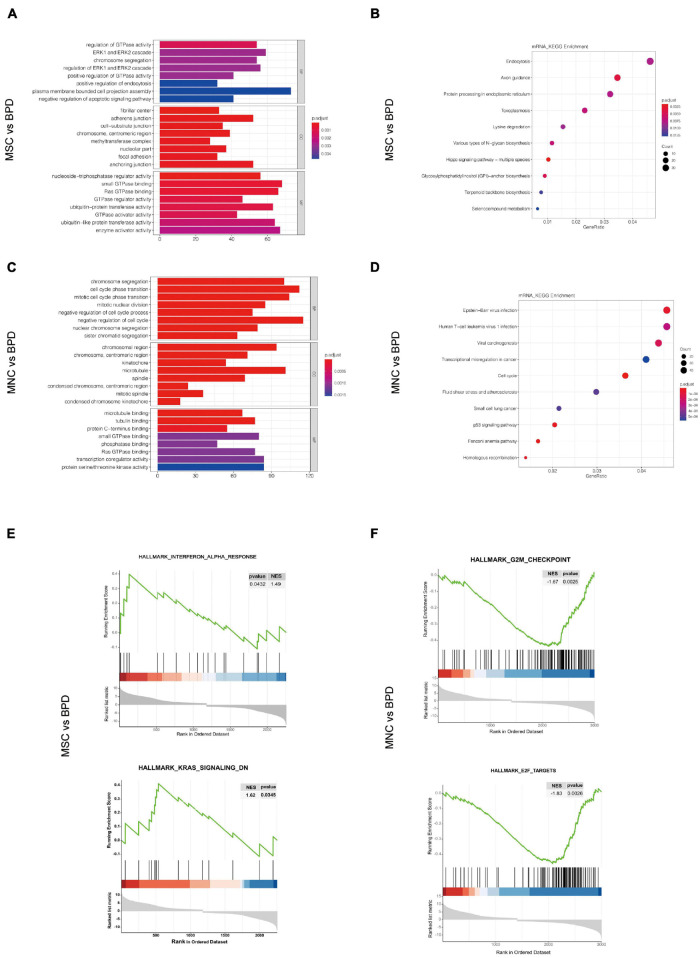
Pathway analysis for the differentially expressed mRNAs (DEmRNAs). **(A,B)** Gene Ontology (GO) analysis of the significantly differently expressed mRNAs in umbilical cord-derived mesenchymal stem cell (UC-MSC)-infused **(A)** and cord blood-derived mononuclear cell (CB-MNC)-infused bronchopulmonary dysplasia (BPD) mice **(B)** relative to BPD mice. **(C,D)** Kyoto Encyclopedia of Genes and Genomes (KEGG) analysis significantly differently expressed mRNAs in UC-MSC-infused **(C)** and CB-MNC-infused BPD mice **(D)**. **(E,F)** Enrichment plots of gene expression signatures for topmost signaling hallmarks by gene set enrichment analysis (GSEA) of DEmRNAs in UC-MSC-infused group and CB-MNC-infused group.

Considering that the complexities of the interactive pairs in lncRNA–miRNA–mRNA networks or the circRNA–miRNA–mRNA networks were both derived from the DEmRNAs in the indicated groups, we firstly performed the GO and KEGG pathway analysis upon DEmRNAs involved in the two different treatments. By conducting GO analysis, 274 GO terms were significantly enriched in DEmRNAs between the UC-MSC-infused and BPD mice, which are mainly related to regulation of GTPase activity (biological_process, GO: 0043087), fibrillar center (cellular_component, GO: 1901673), and nucleoside triphosphatase regulator activity (molecular_function, GO: 0060589). KEGG pathway analysis revealed the top 10 enriched pathways in these DEmRNAs. Of them, endocytosis, Axon guidance and protein processing in endoplasmic reticulum regulation of actin cytoskeleton, and phosphatidylinositol 3-kinase (PI3K)–Akt signaling pathway were the most significantly enriched (Figure 8B). Intriguingly, the introduction of CB-MNCs gives birth to quite different outcomes of pathway enrichment. Among the 823 significantly enriched GO terms between the CB-MNC-infused and BPD mice, the topmost enriched terms are regulation of chromosome segregation (biological_process, GO: 0007059), chromosome region (cellular_component, GO: 0098687), and microtube binding (molecular_ function, GO: 0008017). KEGG pathway analysis revealed the top 10 enriched pathways in these DEmRNAs (Figure 8D). Of them, Epstein-barr virus infection, viral carcinogenesis, and cell cycle signaling pathway were the most significantly enriched.

We also conducted gene set enrichment analysis (GSEA) to better understand the signatures of these significantly changed genes. As shown in revised Figures 8E–F, the hallmarks of the top 2 significantly changed genes in UC-MSC group with *p*-value < 0.05 are interferon_alpha_response (NES = 1.49, *p* = 0.0432), Kras_signaling_DN (NES = 1.62, *p* = 0.0345). While the hallmarks of the significantly changed genes in CB-MNC group with *p*-value < 0.05 are G2M_checkpoint (NES = −1.67, *p* = 0.0025) and E2F targets (NES = −1.83, *p* = 0.0026). The opposite correlation value in the pivotal ways of UC-MSC- and CB-MNC-infused groups was a reflection of the distinct traits of the above two cells. Detailed gene enrichments are listed in Supplementary Tables 5–7. Normalized RNA-seq results of the topmost changed mRNAs, miRNAs, lncRNAs, and circRNAs and the key genes residing in the topmost enriched pathways regarding the divergence of CB-MNC and UC-MSC infusion were shown in Supplementary Figure 6.

### Construction of DEmRNAs Mediating Protein–Protein Interaction Network in Stem Cell-Infused Bronchopulmonary Dysplasia Mice vs. Bronchopulmonary Dysplasia Mice

By converging the four common methods (DEseq2, edgeR, limma-voom, and limma-trend) in analyzing the DEmRNAs between BPD mice and the indicated stem cell-infused mice, 66 DEmRNAs coexist in all analyzed methods between UC-MSC-introduced mice and BPD mice (Supplementary Figure 6A), whereas 330 mRNAs were simultaneously contained in DEmRNAs between CB-MNC-introduced mice and BPD mice (Supplementary Figure 6B). We next constructed DEmRNAs mediating protein–protein interaction networks in indicated groups to reveal their complex interactions among each other using STRING system. Based on the filter parameters of the cytoHubba plug-ins, a total of 25 proteins and 41 edges were included in the network of UC-MSCs vs. BPD group (Supplementary Figure 6C), and a total of 168 proteins and 2,027 edges were included in the network of CB-MNCs vs. BPD group (Supplementary Figure 6D). Furthermore, a Cytoscape MCODE plug-in system was used to identify the key hubs of these PPI networks. The top key regulated genes mediating PPI networks of UC-MSC vs. BPD as well as CB-MNC vs. BPD were shown in Supplementary Figures 6E,F. Module 1 included five DEGs and 10 edges (Supplementary Figure 6E), and module 2 included 53 DEGs and 1,090 edges (Supplementary Figure 6F).

### Construction and Analysis of the Dysregulated mRNA–lncRNA ceRNA Network and Dysregulated mRNA and circRNA ceRNA Network

**FIGURE 9:**
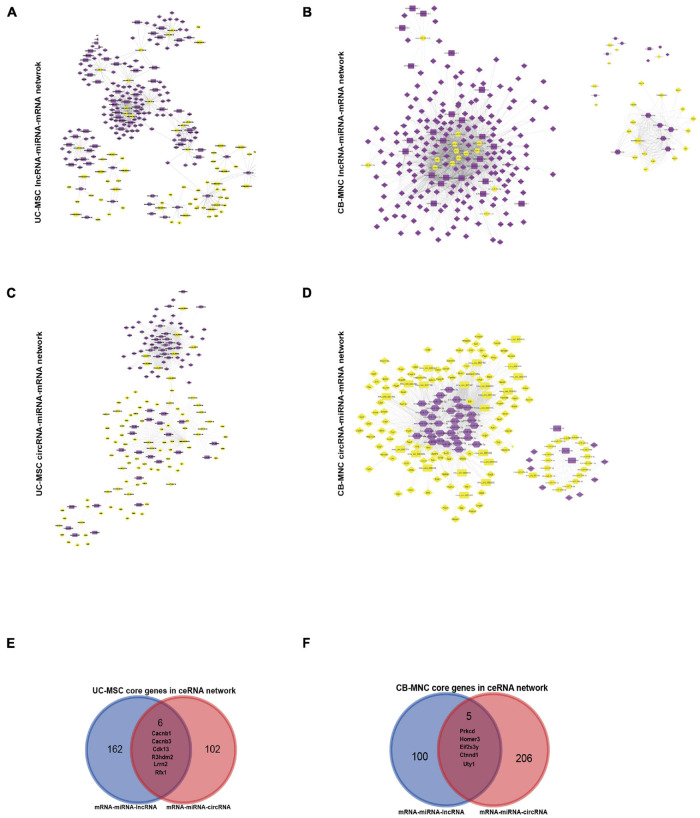
Construction of dysregulated mRNA–lncRNA coexpression network (DMLCN) and the dysregulated mRNA–circRNA coexpression network (DMCCN) based on the correlation value. **(A,B)** The layout of dysregulated mRNA–lncRNA ceRNA network (DMLCN). A total of 239 nodes and 602 edges were identified in umbilical cord-derived mesenchymal stem cell (UC-MSC)-infused groups relative to bronchopulmonary dysplasia (BPD) mice, and a total of 264 nodes and 2,138 edges were identified in cord blood-derived mononuclear cell (CB-MNC)-infused groups relative to BPD mice. Purple diamond: upregulated mRNAs, yellow diamond: downregulated mRNAs; purple hexagon: upregulated miRNA, yellow hexagon: downregulated miRNA; purple round rectangle: upregulated lncRNAs, yellow round rectangle: downregulated lncRNAs. **(C,D)** The layout of dysregulated mRNA–circRNA ceRNA network (DMCCN). A total of 177 nodes and 1,326 edges were identified in UC-MSC-infused groups relative to BPD mice, and a total of 182 nodes and 1,942 edges were identified in CB-MNC-infused groups relative to BPD mice. Purple diamond: upregulated mRNAs, yellow diamond: downregulated mRNAs; purple round rectangle: upregulated circRNAs, yellow round rectangle: downregulated circRNAs. **(E)** The Venn diagram could demonstrate that six key genes were contained in the mRNA–miRNA–lncRNA and mRNA–miRNA–circRNA ceRNA network of UC-MSC-infused group simultaneously. **(F)** The Venn diagram could demonstrate that five genes were contained in the mRNA–miRNA–lncRNA and mRNA–miRNA–circRNA ceRNA network simultaneously.

In brief, firstly, we mapped all the DE-RNAs to the expression matrix in different categories and retrieved the expression profile, then we used miRanda as well as Targetscan to establish the putative miRNA–targets by screening the miRNA-binding sequence. As for the dysregulated mRNA–lncRNA ceRNA network (DMLCN), a candidate lncRNA–mRNA pair was generated if they competed for at least three common miRNAs. Furthermore, positive co-expression pairs between the differentially expressed lncRNA or circRNA and mRNA with coefficients > 0.8 and the negative regulatory relationships between the differentially expressed lncRNA or circRNA and miRNA with coefficients < −0.9 were retained. Finally, all the mRNA–miRNA–lncRNA ceRNA pairs were identified by performing hypergeometric test at the threshold of *p*-value < 0.05. As a result, DMLCN between MSC and BPD groups contained 60 lncRNA nodes (43 upregulated, 17 downregulated), 152 mRNA nodes (111 upregulated, 41 downregulated), 27 miRNA nodes (14 upregulated, 13 downregulated) in 602 pathway edges (Figure 9A). DMLCN between MNC and BPD groups contained 28 lncRNA nodes (25 upregulated, three downregulated), 211 mRNA nodes (193 upregulated, 18 downregulated), and 25 miRNA nodes (10 upregulated, 15 downregulated) in 2,138 pathway edges (Figure 9B). Thereafter, we constructed the dysregulated mRNA and circRNA ceRNA network (DMCCN) by above strategies. Notably, the estimated miRNA–circRNA interactions were obtained by mapping downloaded data in a combination of circBase and Starbase. DMCCN between MSC and BPD mice contained 30 circRNA nodes (eight upregulated, 22 downregulated), 111 mRNA nodes (42 upregulated, 69 downregulated), and 36 miRNA nodes (17 upregulated, 19 downregulated) in 1,326 pathway edges (Figure 9C). DMCCN between MNC and BPD groups contained 25 circRNA nodes (four upregulated, 21 downregulated), 105 mRNA nodes (96 upregulated, nine downregulated), and 52 miRNA nodes (33 upregulated, 19 downregulated) in 1,942 pathway edges (Figure 9D). All of the circRNA/lncRNA–miRNA–mRNA ceRNA pairs were provided in Supplementary Table 6. Importantly, the six DEmRNA nodes that presented in both the mRNA–miRNA–lncRNA and mRNA–miRNA–circRNA ceRNA networks in UC-MSC-implanted BPD group were *Cacnb1* (calcium voltage-gated channel auxiliary subunit beta 1, downregulated, modulator of G protein inhibition) (Lu et al., 2010); *Cacnb3* (calcium voltage-gated channel auxiliary subunit beta 3, upregulated, inducible responder of dendritic cells) (Bros et al., 2011); *Cdk13* (cyclin-dependent kinase 13, upregulated, regulator of global RNA polymerase) (Fan et al., 2021); *Lrrn2* (leucine-rich repeat protein 2, neuronal, upregulated, controller of cell adhesion and movement) (Haines et al., 2005); *R3hdm2* (R3H domain-containing protein 2, downregulated, independent risk factors of cardiovascular system) (Yang et al., 2010); and *RFX1* (regulatory factor RFX1, downregulated, transcriptional factor of FGF1) (Hsu et al., 2012; Figure 9E). On the other hand, five mRNA nodes that presented in both the mRNA–miRNA–lncRNA and mRNA–miRNA–circRNA ceRNA networks in CB-MNC-implanted BPD group were *Prkcd* [protein kinase C, delta, downregulated, suppressor of autophagy (Zhang et al., 2017)]; *Homer3* (Homer protein homolog 3, upregulated, scaffold of neutrophil polarity and involved in GTPase signaling) (Wu et al., 2015); *Eif2s3y* (eukaryotic translation initiation factor 2 subunit 3, Y-linked, downregulated, extracellular signal-regulated kinase (ERK) pathway-dependent regulators in proliferation of spermatogonial stem cells) (Zhang et al., 2021); *Uty* [ubiquitously transcribed tetratricopeptide repeat gene, Y chromosome (downregulated, site-specific histone demethylase (Shpargel et al., 2012)]; and *Ctnnd1* (cadherin-associated protein, delta 1, downregulated, promoters of proliferation in multiple cancers) (Castillo et al., 2010; Figure 9F). To sum up, the key hub DE protein-coding genes along with the ncRNA network can partially explain the therapeutic divergence upon UC-MSC and CB-MNC manipulation.

## Discussion

Hyperoxia causes direct injury to cells mainly through recruiting inflammatory cells to the residential organs, and human MSCs have been disclosed to show enhanced therapeutic effects *via* paracrine secretion or cell-to-cell contact that modulates inflammatory functions or differentiation capacities. Meanwhile, accumulating evidence has suggested that human MSCs can also possess the potential to facilitate tissue repair and stimulate lung maturation (Laube et al., 2016), while limited preclinical experiments have demonstrated that UC-MSCs can improve outcomes under hyperoxia-induced BPD. These might be due to the higher level of quality control and release criteria for UC-MSCs in neonatal manipulation than that of the utility in adulthood as well as the inadequate establishment of general standards in assessing ways of delivery. Considering the side effects of metastatic potential and EMT property raised by human MSC implantation in tumor treatment (Yan et al., 2021), MNCs are optimal alternatives in stem cell-based therapies, especially CB-MNCs, among which we have witnessed an increasing body of data (Yin et al., 2015; Shin et al., 2016). Additionally, the MNCs preferentially resided in the pulmonary concomitant with very low engraftment rate (O’Reilly and Thebaud, 2015). Moreover, unlike the frequently reported applications of UC-MSCs in managing pulmonary dysfunctions, few studies have examined the translational potential of CB-MNCs in neonatal BPD animal models to date, and conclusions remain staggering under different situations (Abreu et al., 2013; Monz et al., 2013; Mills, 2017). In our current research, we demonstrate that intravenous administration of CB-MNCs to hyperoxia-exposed mice had significant effects on stimulating alveolarization, promoting tissue repair, and alleviating pro-inflammatory responses with substantial evidence (Figure 3 compares BPD group with CB-MNC group). Strikingly, CB-MNC transplantation exhibited strong capacity of enhancing the overall lung motion function and maintaining pulmonary–vascular flow homeostasis (Figure 5). Moreover, we also compared the efficiency between independent CB-MNC and UC-MSC infusion in all aspects. We observed generally better (albeit not consistent in all parameters or indices) effects of CB-MNC transplantation in balancing the inflammatory responses and restoring the lung morphogenesis compared to UC-MSC administrations (Figures 3–5).

Over the past few decades, the development of high-throughput sequencing has led to the identification of several lncRNAs and circRNAs in various tissues and cells (Hua et al., 2019; Wei et al., 2019). Increasing evidence has shown that these ncRNAs could participate in the regulation of multiple layers of the pathological and physiological event, including cell proliferation, migration, and metastasis (Zhou S.Y. et al., 2020) through regulating gene expression at the levels of pretranscription, transcription, and posttranscription (Zhang et al., 2019). Despite the combinative analysis integrating multiple datasets from public Gene Expression Omnibus (GEO) database (Wang et al., 2019), comprehensive expression profile and analysis of mRNAs, miRNA, lncRNA, and circRNA with regard to mouse BPD model with or without intervention treatment are vague. To the best of our knowledge, this is the first comprehensive high-throughput sequencing analysis of circRNA, miRNA, lncRNA, and mRNA expression profiles in comparison between the MSC- or MNC-infused BPD mice and BPD mice. We found that there were 2,256 and 2,997 mRNAs, 1,065 and 1,408 lncRNAs, 48 and 58 circRNAs, and 42 and 69 miRNAs with significantly different variations (fold change > 2 and p < 0.05) in UC-MSC and CB-MNC infusions, respectively (Figure 7, Table 2, and Supplementary Tables 1–4). We further navigate to the comparison analysis on the biological pathways of GO (GO-BP) that features the functions of differentially expressed genes. Interestingly, the top GO-BPs in UC-MSC-infused groups are related to GTPase activity (i.e., *Rapgef3*/*Rapgef6*/*Rasgrp1*/*Rap1gap*), ERK1 and ERK2 cascade (i.e., *Fn1*, *Dab2*, *Fgfr2*, and *Fgf3*), and chromosome segregation (Nusap1, Bub1, Map10, and Cenpc1). On the other hand, the top GO-BPs in CB-MNC-infused groups are cohesively related to mitotic nuclear division (*Ccne2*, *Ccn*b1, *Cdc6*, and *Cdc27*), cell cycle phase transition (*Cdk4*, *Cdc25c*, *Aurkb*, and *Ccnd3*), and chromosome segregation (Bub1, Psrc1, Bub1, and Cenp1). Collectively, chromosome segregation a fundamental cell activity, were both drastically changed upon UC-MSC and CB-MNC infusion, which might be an explainable output owing to the strong capacity of stem cells with the key genes varied in different groups. Also, the divergent GO-BPs in the two groups are sense-making, since the stimulating and proliferation effects are robust in CB-MNC-infused BPD, while the widely accepted UC-MSC introduction in alleviating BPD can relieve the injured pulmonary microenvironment from stress suffering by refreshing the motor sensing (GTPase activity) and mitogen activation pathways (ERK1 and ERK2 cascade) (Figure 8).

The pathogenesis of BPD is a complex process characterized by fewer and larger simplified alveoli, influx of inflammatory cells, and endothelial and epithelial cell death (Choo-Wing et al., 2013) and regulated by signaling-regulatory networks (Zhao et al., 2014; Liu et al., 2020; Zhang et al., 2020), including growth factors, transcription factors, regulating enzymes, and ncRNAs, a number of which formed feedback loops controlling the process of damage repair, alveolarization revitalization, and angiogenesis restoration. By combining initial screening of DEmRNA with GO/KEGG analysis, multiple significantly dysregulated genes have drowned consistent conclusion with current studies. For instance, *Semaphorin* 4A is reported to regulate angiogenesis through modulating the VEGF pathway (Segarra et al., 2012), and *Ucp2* is a macrophage-specific inducer in response to pulmonary injury (Wang et al., 2016). Besides, many studies have indicated the important roles of an inflammatory response and immune response in cancers (Vilariño et al., 2020) and neurodevelopment (Tan et al., 2020). Among the gene lists of the top 10 most significantly upregulated and downregulated genes interfered by stem cell implantation, *NXT2* played pivotal roles in neurodevelopment, whereas *Mknk2*, *Sema6a*, and *PIR2* are frequently dysregulated molecules in the pathogenesis of multiple cancer types as evidenced by GeneRIF documented by PubMed. While implantation of UC-MSCs or CB-MNCs expectedly redirected the expression pattern (refer to Table 1 and Supplementary Tables 1–4) of these candidates, further verifications are important for deepening the understanding of BPD and lay a good prediction ability for evaluating the treatment effects through single-factor model or multiple-factors model.

Despite their poor conservation and low levels of expression compared with protein-coding genes, lncRNAs are often regulated by transcription factors and are expressed in a cell- or tissue-specific manner. In this study, most of the lncRNAs in the co-expression network were not yet annotated. In addition, from DMLCN, the lncRNA–mRNA co-expression network, we found that *Rmi2* coexpressed with most numbers of lncRNAs (Figure 8A) and *Eif2s3y* coexpressed with most numbers of lncRNAs (Figure 8B), forming a complex network in UC-MSC-infused group or CB-MNC-infused group relative to BPD model, respectively. More importantly, recent studies have revealed that these hub genes played pivotal roles in pulmonary malfunctions (Ulke et al., 2019). It is therefore very much worth to perform further studies to reveal the underlying mechanisms of these lncRNAs and the interactions with the guiding hub genes.

Evidence is emerging that circRNAs can participate in the regulation of gene expression in various ways. It has been reported that circRNAs can function through their parental genes (Xu et al., 2020). Additionally, many more circRNAs have been reported to harbor multiple miRNA-binding sites, which seem to be a typical feature of this class of RNA molecules. This feature, together with covalently closed loop structure, suggests that circRNA can act as a sponge of miRNA to regulate a myriad of target genes. Interestingly, the DMCCN network we built exhibited a much concentrated network with highly centered distribution of the circRNA nodes in both UC-MSC- and CB-MNC-related groups compared with the scattered lncRNA distribution (Figures 9C,D). Intriguingly, much progress has been achieved in elucidating the roles of circRNA in lung cancer and multiple adult pulmonary malfunctions. For instance, a novel circular RNA, circXPO1, promotes lung adenocarcinoma progression by interacting with IGF2BP1 (Huang et al., 2020), and hot-star circRNA CDR1as and CircRNA0001859 are key players in balancing cardiovascular–pulmonary homeostasis (Chen S. et al., 2020; Ma et al., 2020). Linking the novel discovered circRNAs in alleviating BPD and stem cell-based therapy will make them optimal biomarkers.

Of note, we interpret the overall prior phenotype in CB-MNC group compared to UC-MSC group in two aspects: one is the same gene, same variation trend with different expression fold changes that impacts the regulation level and the other are distinct genes involved in the same pathway with divergent regulation modes. We focused on the significantly changed genes that reside in the hallmark pathways by GSEA analysis and BP term of GO analysis, among which *Clic3* (chloride intracellular channel protein 3), *Kdm4b* (lysine demethylase 4B), *Pxn* (paxillin), *Sorbs2* (sorbin and SH3 domain-containing protein 2), *Trafd1* (TRAF-type zinc finger domain containing 1), and *Tsku* (Tsukushi) coexisted in both CB-MNC and UC-MSC groups with larger extent variations in CB-MNC groups (Supplementary Table 7, sheet co_GSEA). As typical examples, their geneRIFs are closely related in the cellular hallmarks of BPD including epithelial transition, endothelial homeostasis, and inflammatory responses (Mashima et al., 2005; Zhou S. et al., 2020; Hurskainen et al., 2021). Tsku is a small leucine-rich proteoglycan that has been documented to be induced in proteomic profiling of a TGF-β1-induced *in vitro* model of fibrosis in rat kidney fibroblasts (Zhou S. et al., 2020). When we reviewed our data, MSC group: downregulated log_2_FC −4.99, MNC group: −7.50, implying that introduction of the indicated stem cell can revert the induced Tsku level and the level of TGF-β expression with distinct extent. Meanwhile, in lung cancer cells, TSK expressed more highly than the other small leucine-rich repeat proteoglycan family members and regulates the epithelial–mesenchymal transition and cell proliferation (Yamada et al., 2019; Huang et al., 2021), indicating its pivotal functions in reverting the abrogated epithelium development and retarded EC proliferation. SORBS2 is another example of epithelial-regulating molecule, as a component of the acto-myosin ring at the apical junctional complex in epithelial cells (Fredriksson-Lidman et al., 2017) SORBS2 is a scaffolding protein associated with Abl/Arg non-receptor tyrosine kinase pathways and is known to interact with actin and several other cytoskeletal proteins in various cell types. The downregulated SORBS2 expression in both groups (CB-MNC: log_2_FC −6.25, UC-MSC: log_2_FC −6.52) implies that the rebalancing of the epithelial junction complex greatly contributes to the integrity of the pulmonary development. Another interesting example is Pxn (paxillin), whose knockdown has been proven to enhance endothelial cell migration *in vitro* and stimulate angiogenesis during normal development and in response to tumor angiogenic factors *in vivo* (German et al., 2014). In our data, MNC group pxn attenuated at Log_2_FC −9.88 while MSC group pxn reduced at log_2_FC −5.50, indicating the angiogenesis promoting functions of this gene in rescuing histopathological phenotype of abnormal growth and development. We also found that Clic3 is a chloride intracellular channel protein. It has been reported that its homologous family member, Clic1, has been significantly reduced in severe BPD compared to the moderate BPD (Magagnotti et al., 2013). Infusion of CB-MNC robustly enhanced the expression level of Clic3, with log_2_FC 5.06 in UC-MSC group while log_2_FC 9.88 in CB-MNC group. The calcium-related channel protein might participate in the reescalating effect of CB-MNC and UC-MSC in alleviating BPD.

We have thoroughly detected the factors regarding inflammatory regulation, tissue repair, and vascular remodeling (Figure 4). Interestingly, the classical pro-inflammatory factors (i.e., IL-6 and IL-1β) and classical anti-inflammatory factors (i.e., IL-10 and IL-2) exhibited a Yin–Yang balance expression module in response to experimental BPD model expectedly (Figures 4A–E). Inflammation is generally considered to be detrimental in recovery from hyperoxia-induced lung injuries, while single use of anti-inflammatory treatments targeting specific inflammatory mediators has yet been ineffective to date. It has been proven that Toll-like receptor (TLR)-mediated regulation of inflammasomes is a significant prognostic marker of BPD with different severities (Liao et al., 2015; Syed et al., 2019). Furthermore, cell-extrinsic responses induced by TLR signaling consist of inflammation (TNF-α) and tissue repair (IL-10). In the GSEA-enriched core genes, Trafd1, also named FLN29, is a novel interferon- and lipopolysaccharide (LPS)-inducible gene acting as a negative regulator of TLR signaling. As shown in Figures 4A–E, restraint levels of inflammatory marker IL-1β and IL-6 were concomitant with augmenting secretion of IL-10 and TGF-β. The reversion effect was more significant in CB-MNC group than in UC-MSC group (with log_2_FC in CB-MNC 6.72 and log_2_FC in UC-MSC 1.52). The unveiled functions of Trafad1 might provide another great example in illustrating the conversion of the inflammatory network.

The slightly declined TGF-β signaling and MMP-9 expression along with escalated VEGF expression after CB-MNC infusion (Figures 4F,G) implied the prevalence of adopting MNC in both balancing the delicate and intertwining feature of inflammatory networks and rescuing the devastative developing lung in experimental BPD model caused by hyperoxia exposure. The recovery of an intact epithelium following lung injury is critical for restoration of lung homeostasis, which includes an acute inflammatory response, recruitment of immune cells, and epithelial cell spreading and migration upon an autologously secreted provisional matrix. MMP-9 involves the breakdown of extracellular matrix in normal physiological and pathological processes regarding pulmonary homeostasis. Several key signaling pathways are important in regulating these processes, including sonic hedgehog, Rho GTPases, MAP kinase pathways, STAT3, and Wnt (Crosby and Waters, 2010), within which the uniquely differently expressed hub genes reside in the pathways (Supplementary Table 7). Previous studies have established the functional links between oxidative stress, apoptosis, autophagy, and endoplasmic reticulum (ER) stress through the nuclear factor erythroid-like 2 (Nrf2)/antioxidant response element (ARE) signaling pathways (Chen Y. et al., 2020; Zhang et al., 2020), and it has been reported that mesenchymal stem cells attenuate diabetic lung fibrosis *via* adjusting Sirt3-mediated stress responses in rats. In our data, in CB-MNC vs. BPD group, Sirt3 was among the significantly changed genes (log_2_FC 6.93, sheet CB-MNC vs. BPD group), while in UC-MSC, the changed Sirt family members were sirt2, and the expression level was downregulated (Chen Y. et al., 2020). Aberrant pulmonary vascular growth and remodeling are frequently seen in bronchopulmonary dysplasia (Alvira, 2016), and the fibroblast growth factor (FGF)-2 and VEGF are promising targets in the treatment of respiratory disorders (Laddha and Kulkarni, 2019). In CB-MNC group, the FGF-2 was slightly decreased (log_2_FC −1.68), and the FGFR (log_2_FC 1.27) was slightly increased (see sheet UC-MSC vs. BPD). This might partly explain the moderately mitigated VEGF level in MSC-introduced groups while the escalating of VEGF in CB-MNC group was largely due to the role of FoxM1, a transcriptional regulator of G1/S and G2/M transition and M phase progression in the cell cycle and a multifaced regulator in pulmonary disease. It can significantly activate adherens junctions, vascular formation, and pulmonary inflammation through multiple direct targets. In our data, the CB-MNC infusion can largely increase the level of Foxm1 (log_2_FC 2.46). Therefore, the robust enhancement of VEGF in CB-MNC group can be explained, which accounts for the second way of understanding the links between key genes in the pathways. Collectively, the concentrated molecules and hub genes are interpretable in understanding the divergence of UC-MSC and CB-MNC in alleviating BPD, especially the phenotypes related with VEGF, MMP-9, TGF-β.

Interestingly, the hub DEmRNAs within ceRNA pairs between UC-MSC and BPD groups are closely related with cell cycle integrity, cell adhesion, cell proliferation, and transcriptional homeostasis. The hub DEmRNAs within ceRNA pairs between CB-MNC and BPD groups are closely related with autophagy, neutrophil polarity, cell proliferation, and histone methylation, which is consistent with the result of global DEmRNA GO analysis. Taken together, our findings may provide new evidence for the underlying mechanisms of mRNA/ncRNAs and related ceRNA networks in stem cell-infused BPD and uncover novel targets for better utilizing stem cells in the treatment of BPD (Figure 1).

## Data Availability Statement

The datasets presented in this study can be found in online repositories. All the datasets of RNA-seq included in this study have been uploaded to the GSA system with GSA ID: CRA004720 and BioProject ID: PRJCA004041 and are publicly accessible at https://ngdc.cncb.ac.cn/gsa/browse/CRA004720 after the release date of December 14, 2022.

## Ethics Statement

Animal procedures were reviewed and approved by the Animal Care and Ethics Committee of the Seventh Medical Center of PLA general Hospital (No. 2020-037).

## Author Contributions

JC, YC, and ZF conceptualized the study and wrote the manuscript. JC, YC, and XD conceived and designed the study. XD and JP contributed to the performance of the mice husbandry, BPD animal model, and pulmonary function test. GL, XZ, XF, and FX participated in stem cell purification and injection. YC, JC, and XD performed gene expression analysis and immunohistochemistry. YC and XD performed the computational analysis of the data. XY and XW contributed to the discussion and polish of the manuscript. All authors read and approved the manuscript.

## Conflict of Interest

GL was employed by the company Shandong Qilu Stem Cell Engineering Co., Ltd. The remaining authors declare that the research was conducted in the absence of any commercial or financial relationships that could be construed as a potential conflict of interest.

## Publisher’s Note

All claims expressed in this article are solely those of the authors and do not necessarily represent those of their affiliated organizations, or those of the publisher, the editors and the reviewers. Any product that may be evaluated in this article, or claim that may be made by its manufacturer, is not guaranteed or endorsed by the publisher.

## Footnotes

1
http://www.geneontology.org

2
https://www.genome.jp/kegg
