# Supplementary material for: Integrative Studies of Human Cord Blood Derived Mononuclear Cells and Umbilical Cord Derived Mesenchyme Stem Cells in Ameliorating Bronchopulmonary Dysplasia
Source: Front Cell Dev Biol. 2021 Nov 9;9:679866. doi: 10.3389/fcell.2021.679866 (PMC8631197; doi:10.3389/fcell.2021.679866)

SUPPLEMENTARY FIGURE 1 Representative H&E staining images of female mice lung histology in hyperoxia exposed mice with indicated treatments at PN28 (a). Scale bar: 100 μm. Sections of whole lungs were analyzed for the number of alveoli per square millimeter, the chord length, and the size of the alveoli (b)

SUPPLEMENTARY FIGURE 2 (a) Immuno-histochemical staining of vascular endothelial growth factor (VEGF), transforming growth factor β1 (TGF-β1) and Matrix metalloproteinase-9 (MMP9) in female mice lung tissue. Positive staining of cytoplasm is yellow-brown and that of nuclei is blue. Scale bars 100 μm. (b) Summary of the quantification of the immune-histochemical staining of the indicated factors in (a).

SUPPLEMENTARY FIGURE 3 (a-e) pulmonary function testing among female mice groups. The indicators include peak inspiratory flow (PIF) (b), peak expiratory flow (PEF)(c), tidal volume (TV)(d), breathing per minute (BPM)(e) and minute volume (MV)(f). Values are mean ± SD of a minimum of six animals in each group,*P < 0.05, **P < 0.01, ***P <0.001, by one way ANOVA test. (f-g) Bar graph showing lung/heart blood flow ratio analysis of pulmonary vascular function in different groups at indicated time points. (f) left side and (g) right side. Values are mean ± SD of a minimum of six animals in each group, *P <0.05, **P <0.01, ***P <0.001, by one way ANOVA test.

SUPPLEMENTARY FIGURE 4 Toxicity test. (a) Bar graph illustrating the change of weight from C57 BL/6J mice with control group and CB-MNCs group at PN 28. (b-f) pulmonary function testing among female mice groups. The indicators include peak inspiratory flow (PIF) (b), peak expiratory flow (PEF)(c), tidal volume (TV)(d), breathing per minute (BPM)(e) and minute volume (MV)(f). Values are mean ± SD of a minimum of six animals in each group,*P <0.05, **P <0.01, ***P <0.001, by one way ANOVA test). (g-h) Bar graph showing lung/heart blood flow ratio analysis of pulmonary vascular function in different groups at indicated time points. (g) left side and (h) right side. Values are mean ± SD of a minimum of six animals in each group, *P <0.05, **P <0.01, ***P <0.001, by one way ANOVA test.

SUPPLEMENTARY FIGURE 5 Bar graph of selected topmost differently expressed

RNAs and topmost pathway enriched DERNAs from RNA-seq (a-b) Log2 Fold change was calculated to present the relative expression profiles of selected DEmRNAs in UC-MSCs infused and CB-MNCs groups conducted by RNA-seq. Log2 Fold change was calculated to present the relative expression profiles of topmost differently changed DElncRNA (c-d), DEmiRNA (e-f) and DEcircRNA (g-h) in UC-MSCs infused and CB-MNCs groups conducted by RNA-seq. Bar graphs present the mean ± standard error.

SUPPLEMENTARY FIGURE 6 Construction of differently expressed mRNAs mediated PPI network in CB-MSCs infused and CB-MSCs infused mice. (a-b) Venn diagram depicting the consistently differently expressed genes analyzed by four distinct method within R packages in CB-MSCs infused and CB-MSCs transplanted mice relative to BPD mice, respectively. Notably, the consensus DEGs were subjected to the following network building. (c-d) The comprehensive PPI networks in CB-MSCs infused and CB-MSCs infused mice relative to BPD mice, respectively. The network of CB-MSCs v.s. BPD consists of 25 nodes and 41 edges (c), the network of CB-MSCs v.s. BPD consist of 168 nodes and 2027 edges. (e-f) The top 2 key modules were identified in CB-MSCs infused and CB-MSCs infused mice relative to BPD mice, respectively. The module 1 included 5 DEGs and 10 edges (e), the module 2 included 53 DEGs and 1090 edges (f).

Supplementary Figure 1

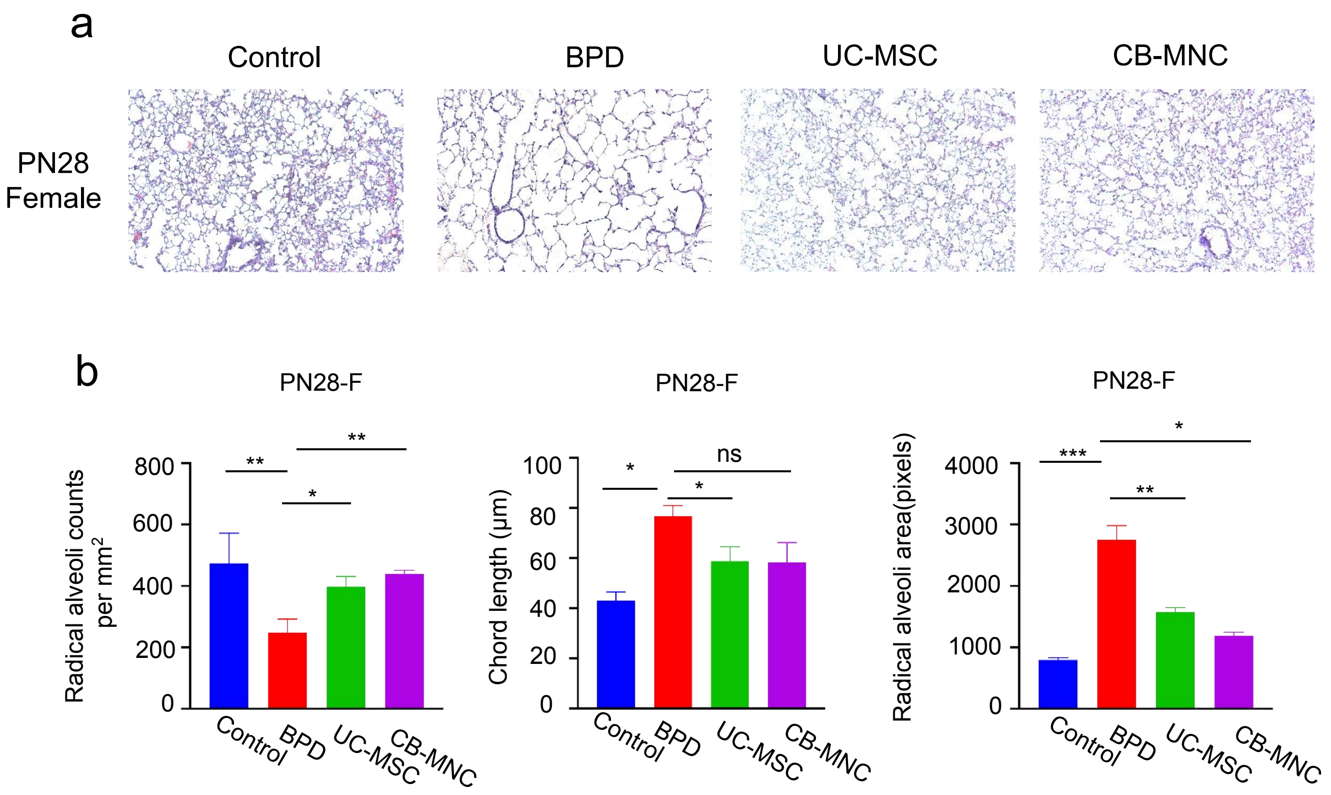

Supplementary Figure 2

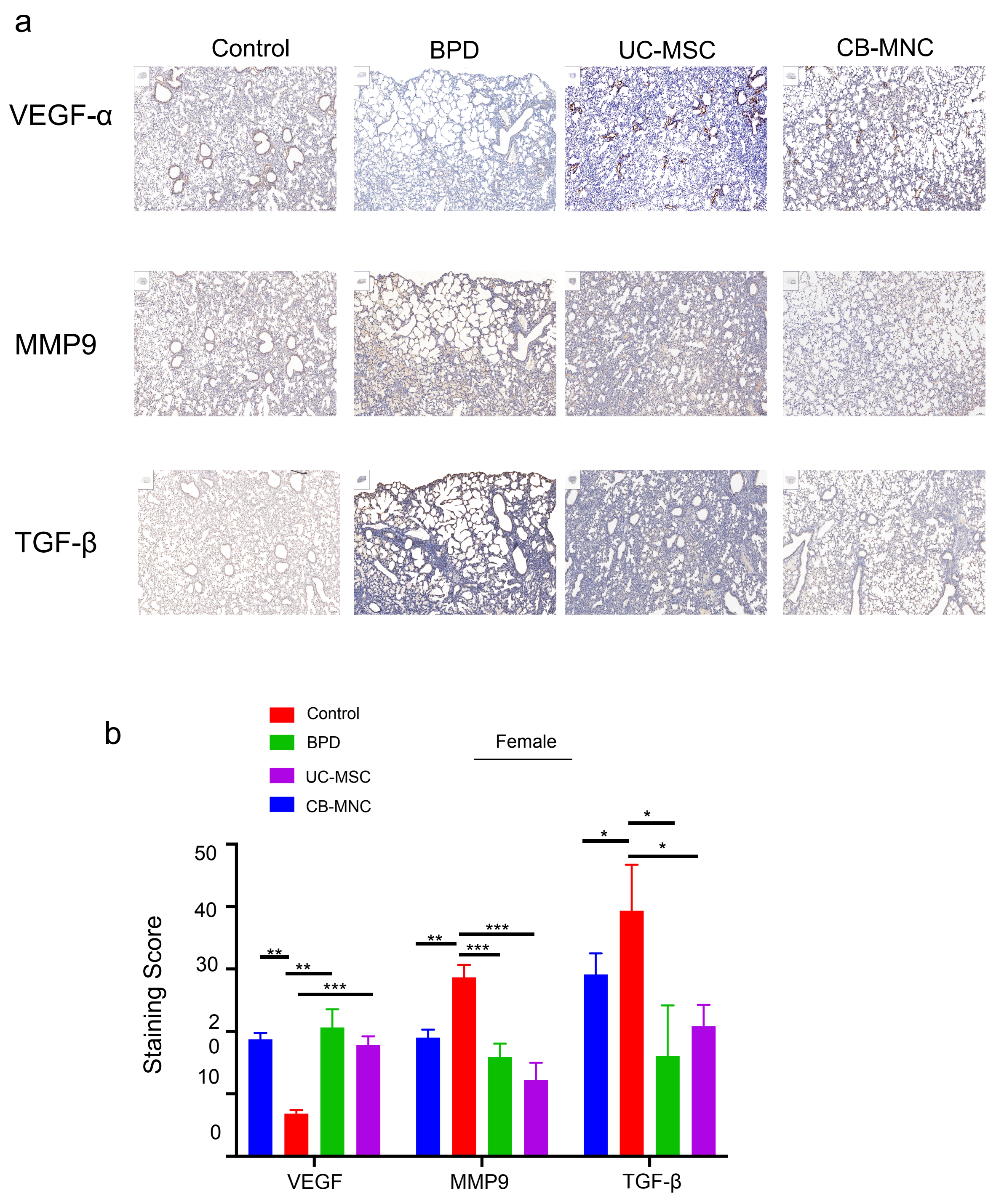

Supplementary Figure 3

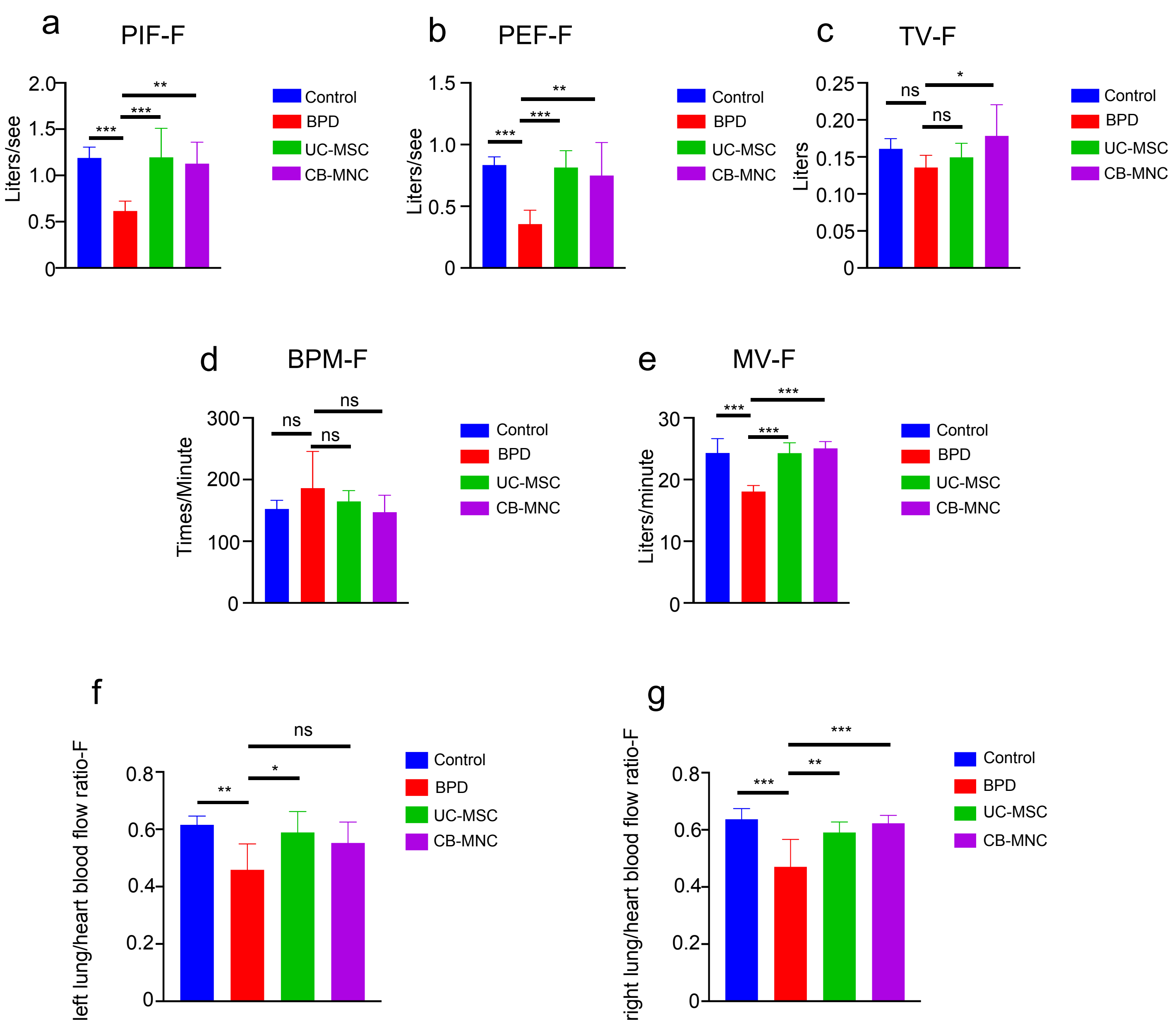

Supplementary Figure 4

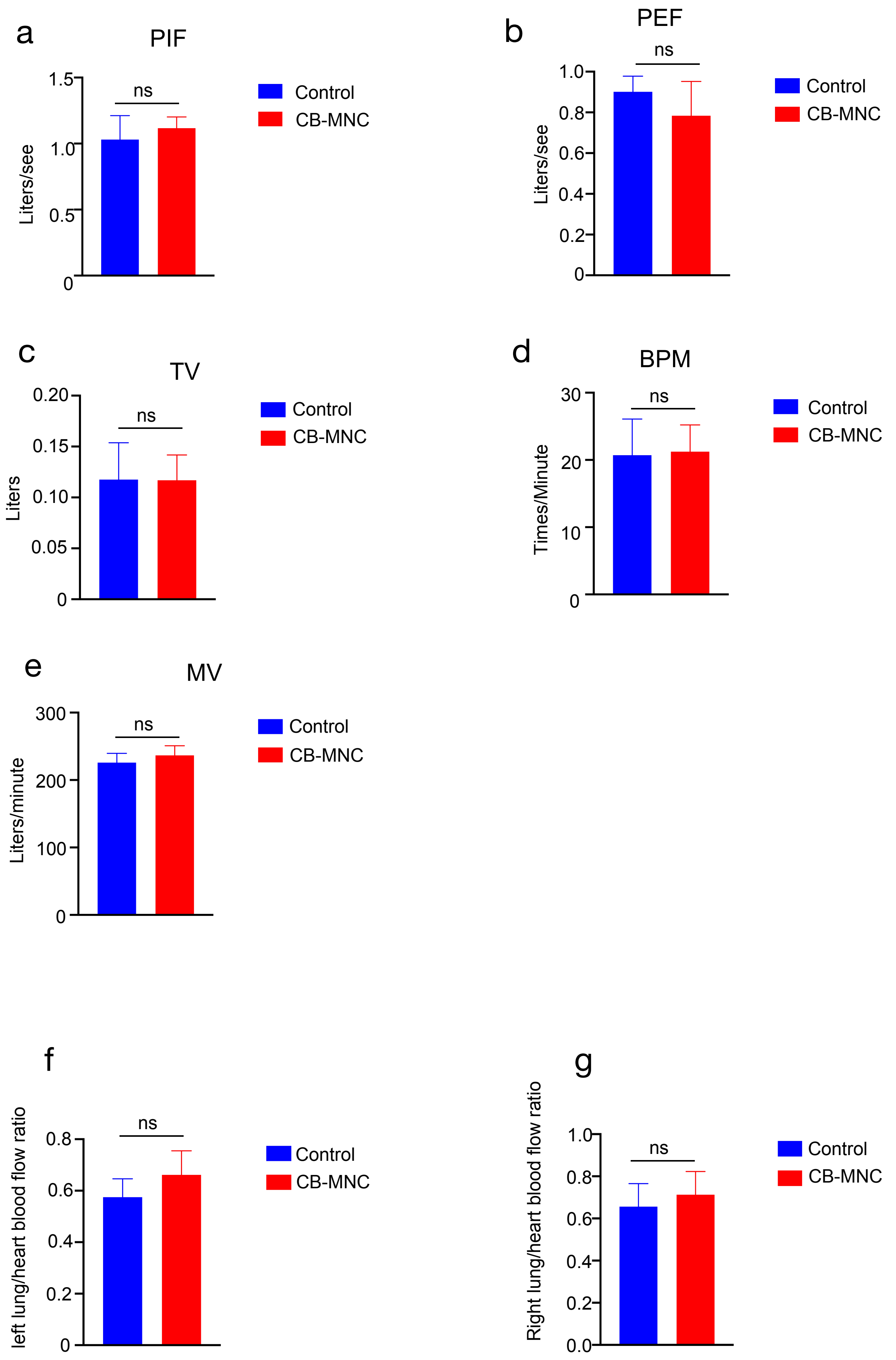

Supplementary Figure1

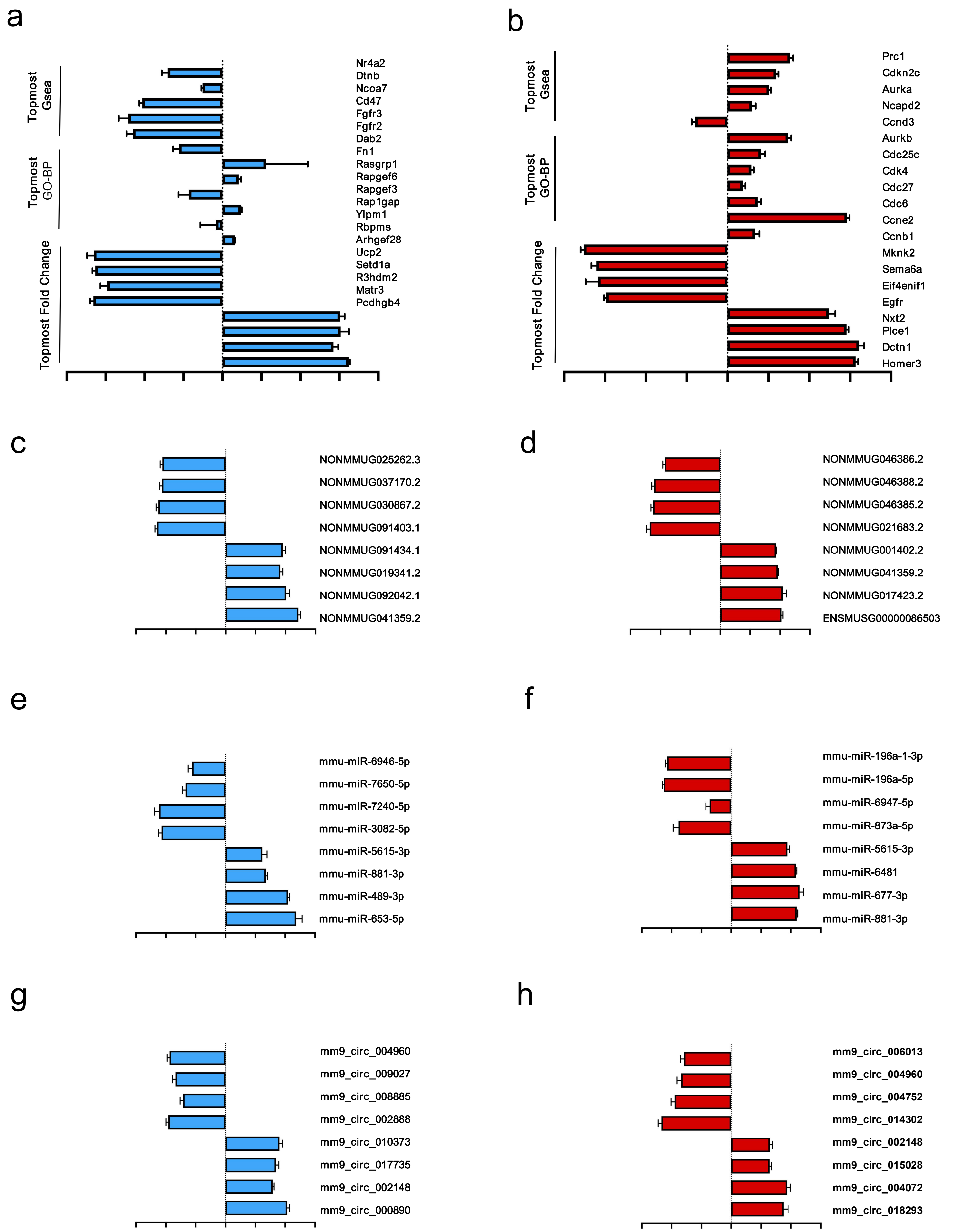

Supplementary Figure 2

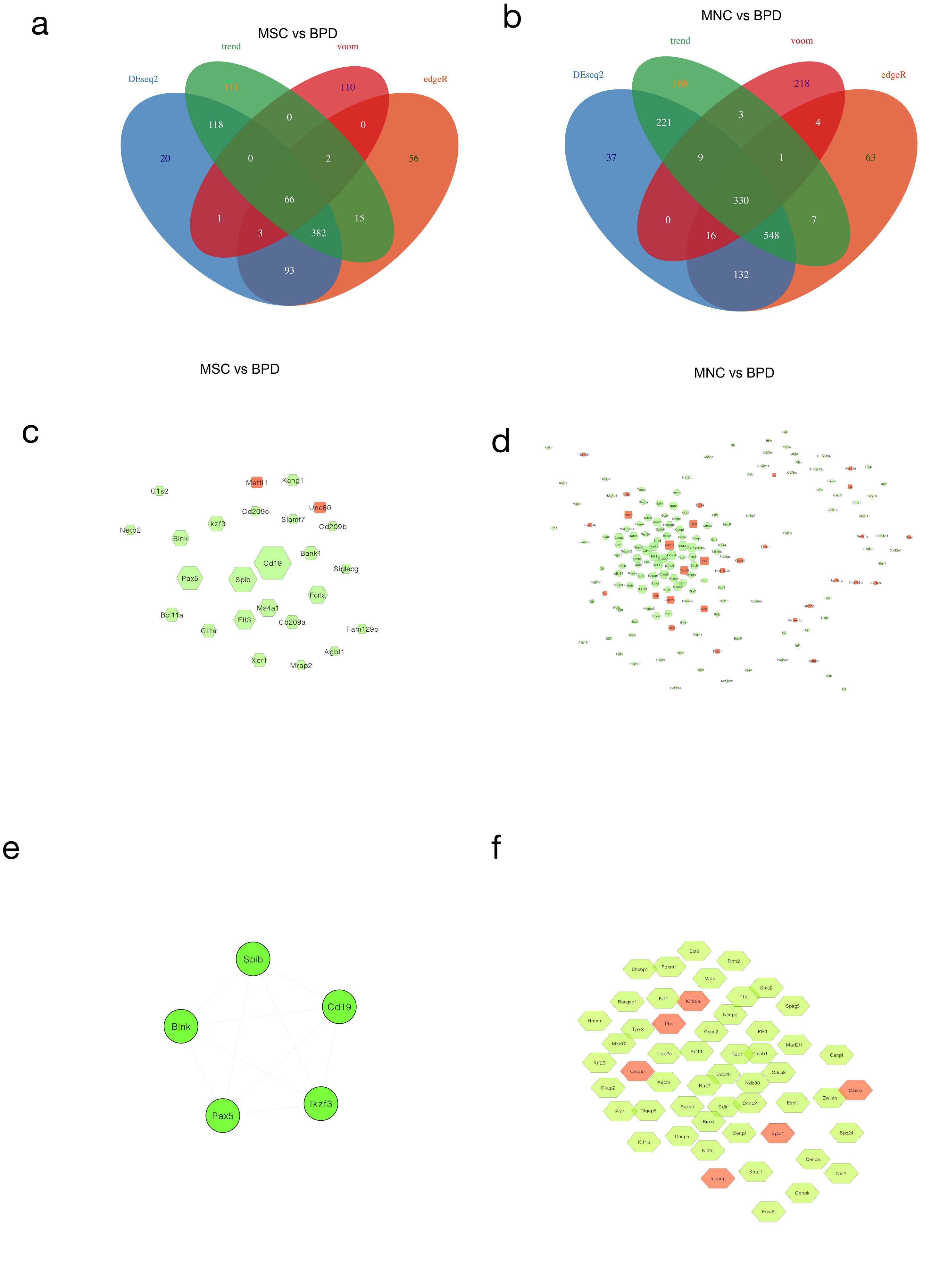

Supplement: Supplementary file 8 [file Data_Sheet_1.PDF]
